# Research progress of near-infrared fluorescence probes based on indole heptamethine cyanine dyes in vivo and in vitro

**DOI:** 10.1186/s13065-020-00677-3

**Published:** 2020-03-30

**Authors:** Chunlong Sun, Wen Du, Baoqin Wang, Bin Dong, Baogui Wang

**Affiliations:** College of Biological and Environmental Engineering & Shandong Key Laboratory of Eco-Environmental Science for the Yellow River Delta & Shandong Provincial Engineering and Technology Research Center for Wild Plant Resources Development and Application of Yellow River Delta, Binzhou University, Binzhou, 256603 China

**Keywords:** Near-infrared fluorescence probes, Indole heptamethine cyanine dyes, Biological application, Research progress

## Abstract

Near-infrared (NIR) fluorescence imaging is a noninvasive technique that provides numerous advantages for the real-time in vivo monitoring of biological information in living subjects without the use of ionizing radiation. Near-infrared fluorescent (NIRF) dyes are widely used as fluorescent imaging probes. These fluorescent dyes remarkably decrease the interference caused by the self-absorption of substances and autofluorescence, increase detection selectivity and sensitivity, and reduce damage to the human body. Thus, they are beneficial for bioassays. Indole heptamethine cyanine dyes are widely investigated in the field of near-infrared fluorescence imaging. They are mainly composed of indole heterocyclics, heptamethine chains, and N-substituent side chains. With indole heptamethine cyanine dyes as the parent, introducing reactive groups to the parent compounds or changing their structures can make fluorescent probes have different functions like labeling protein and tumor, detecting intracellular metal cations, which has become the hotspot in the field of fluorescence imaging of biological research. Therefore, this study reviewed the applications of indole heptamethine cyanine fluorescent probes to metal cation detection, pH, molecules, tumor imaging, and protein in vivo. The distribution, imaging results, and metabolism of the probes in vivo and in vitro were described. The biological application trends and existing problems of fluorescent probes were discussed.

## Introduction

**Fig. 1:**
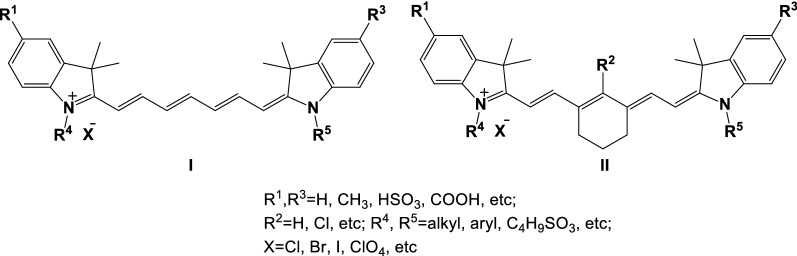
Structures of indole heptamethine cyanine dyes

Indole heptamethine cyanine dyes are the widely used class of cyanine dyes, and the ability to generate strong fluorescence emission at the near-infrared (NIR) region of 650–900 nm [1, 2]. Indole heptamethine cyanine dyes are composed of indole heterocyclic rings, heptamethine chains, and N-substituted side chains (Fig. 1) [3]. In recent years, indole heptamethine cyanine dyes have been widely used in biology; active groups are introduced into indole heptamethine cyanine dyes, or their structural nuclei are altered. The newly generated fluorescent probes have been widely investigated in the field of fluorescence imaging because of their different biological functions. In the present work, the application of indole heptamethine cyanine dyes to the detection of pH changes in vivo and in vitro, trace metal ions, active small molecules, tumor cell-targeted imaging, and other aspects was reviewed to provide references for the development of NIR fluorescence probes in the fields of biological science and medical imaging.

## Main text

### Application of pH-sensitive NIR Fluorescence probes

Organisms have the ability to regulate the acid–base balance in vivo. However, such ability is weakened when organisms are affected by malignant diseases and leads to acid–base imbalance and causes the pH value of body fluids to exceed the normal range [4–6]. Some dyes exhibit different absorption or fluorescence properties with changes in pH values [7–12]. Fluorescence pH measurement showing different optical signals with pH changes can compensate for the deficiency of other methods for pH detection [13, 14]. In addition, the fluorescence method has the advantages of high detection sensitivity, simple operation and portability [15], which is suitable for fluorescence biomolecular studies and for detecting pH changes in cells in real time. The pH fluorescence probes can be divided into pH fluorescence probes for detecting neutral ranges and those for detecting acidic environments. Intracellular acidity is approximately 4.5–6.0, and the pH range of the cytoplasm is approximately 6.8–7.4. Many normal physiological processes in cells and organelles are associated with intracellular pH values [16, 17]. The changes in intracellular pH are associated with muscle spasm, cell proliferation, apoptosis, ion transport, homeostasis, multidrug resistance, malignancies, endocytosis, and Alzheimer’s disease [18]. Therefore, hydrogen ion change is the main research target in vivo, and pH sensitive NIR fluorescence probes provide a new method for effectively monitoring pH changes in vivo.

**Fig. 2:**
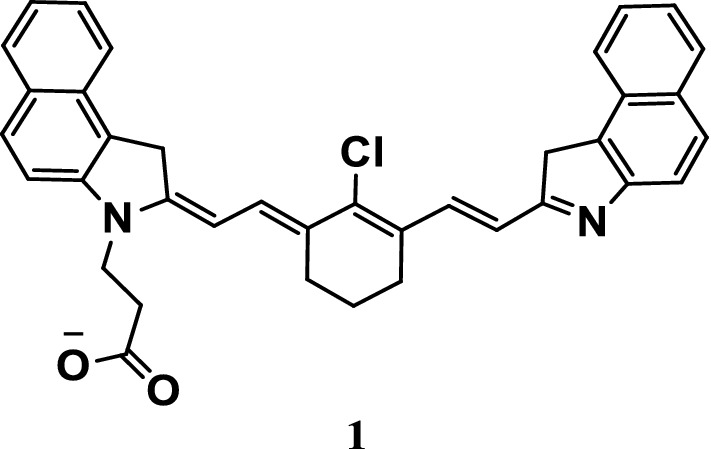
Structure of probe 1

Xue et al. [19] reported a pH-responsive photothermal ablation probe 1 based on cyanine dyes (Fig. 2) for photothermal therapy (PTT), the absorption of which in the NIR was increased by probe 1 by accepting protons. Compared with normal cells, the nanoparticles were formed by bovine serum albumin and probe 1 preferentially accumulate and could be activated in the acidic environment of the Golgi of cancer cells. Moreover, PTT could be effectively conducted in vivo and in vitro.

**Fig. 3:**
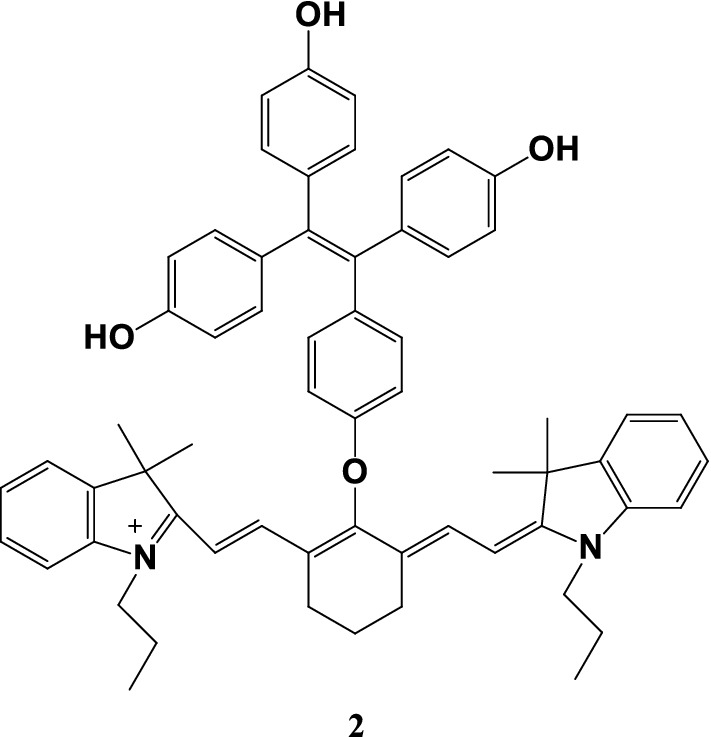
Structure of probe 2

Fang et al. [20] synthesized a fluorescent probe 2 (Fig. 3) using bond-penetrating energy transfer. Probe 2 was consisted of ether bonds formed by tetrastyrene donors and cyanide receptors and exhibited aggregation-induced emission. The probe has dual visible and NIR excitation and emission capabilities, and can detect intracellular pH fluctuations in HeLa cells.

**Fig. 4:**
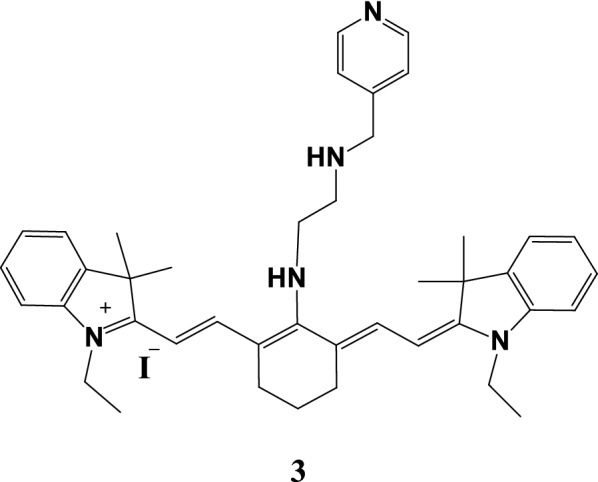
Structure of probe 3

Fang et al. [21] used IR-822 as a fluorescent group because of its excellent tumor preferential aggregation and other characteristics. IR-822 was conjugated to N1-(pyridine-4-methyl) ethane-1,2-diamine (PY), a pH sensing receptor, to form a fluorescent spacer receptor molecular probe 3 (Fig. 4) to enhance its specificity in tumor imaging. The imaging principle is to adjust the fluorescence emission intensity by a fast photoinduced electron to achieve the probe “turn on” under the acidic tumor microenvironment and enhance fluorescent imaging. Probe 3 has a strong absorption capability at 600–850 nm. The probe achieved high spatial resolution photoacoustic (PA) imaging in mice and photothermal ablation of tumors. The mice exhibited significant ablation and no recurrence of tumors following the application of the 808 nm laser irradiation and probe 3 photothermal treatment.

**Fig. 5:**
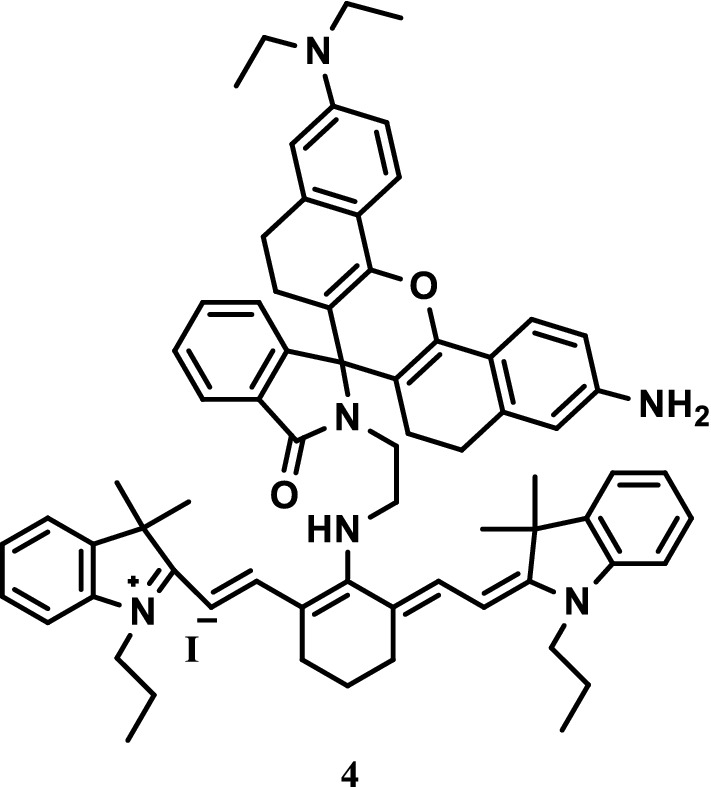
Structure of probe 4

Zhang et al. [22] synthesized a NIR fluorescence probe 4 (Fig. 5) with dual inspection characteristics for pH sensing. The probe was formed by binding a NIR rhodamine donor to a cyanide receptor via a C-N bond with short ethylene suppository. Probe 4 containing the cyanine and rhodamine moieties, showed corresponding fluorescence increases with pH decreases to achieve the double-checked capability, and responded to change in fluorescence intensity under the excitation of the rhodamine body at 450 nm with pH changes.

**Fig. 6:**
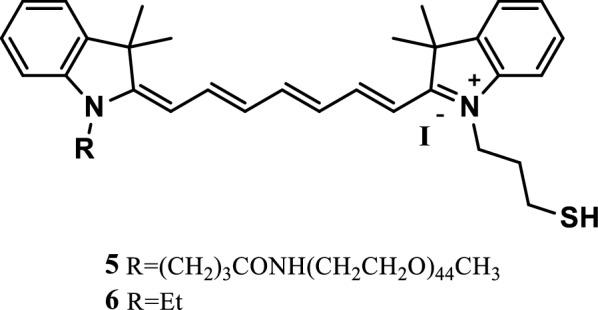
Structures of probes 5 and 6

Mu et al. [23] synthesized two pH-responsive NIR fluorescence probes 5 and 6 (Fig. 6), which could be encapsulated in self-assemblies consisting of anionic, cationic, and neutral amphiphiles in phosphate buffered saline. The self-assembled polyethylene glycol (PEG)-tocopherol conjugated of neutral surfactants effectively encapsulated the dyes and did not lose their pH responsiveness for hours. At the same time, the pH-responsive dyes in self-assembled packaging were sensitive to acidification, the absorption and emission of NIR immensely increased, and the high pH response of self-assembled dyes with non charged surfactants was demonstrated.

**Fig. 7:**
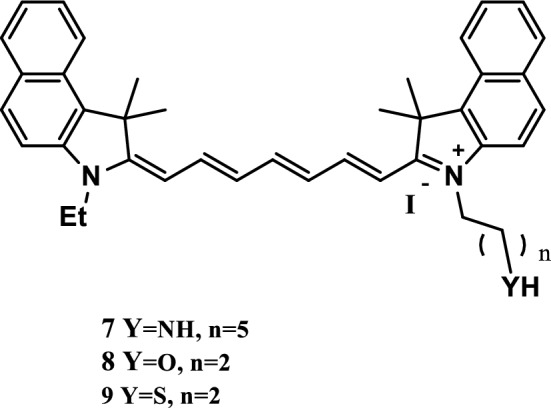
Structures of probes 7, 8, and 9

Koji Miki et al. [24] reported three NIR fluorescence probes 7, 8 and 9 (Fig. 7) that responded to acidity and basicity and were essentially an indocyanine green (ICG) derivative with nucleophilic subunits (amino, hydroxyl, and mercapto). These dyes showed the pH dependence balance between the fluorescent open-loop and nonfluorescent closed-loop structures in the pH ranges of 7–9, 5–7, and 3–6. In vitro culture experiments of HeLa cells showed that probes 8 and 9 exhibited strong pH-dependent fluorescence enhancement through endocytosis. Control incubation experiments at 4 °C showed that the probes positively reacted with the open-loop structure, and ICG combined with the cell membranes through electrostatic interaction. The pH-responsive probes 8 and 9 were found to be powerful probes, which could be used for the highly sensitive analysis of active cells and high-contrast optical imaging of acidic parts of tissues, such as tumors.

**Fig. 8:**
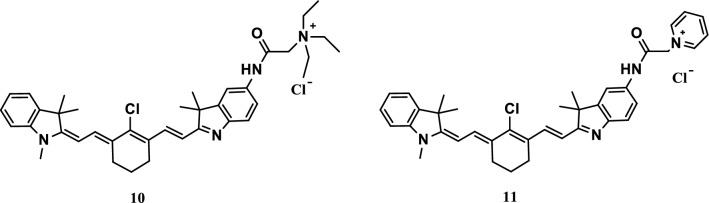
Structures of probes 10 and 11

Hou et al. designed and synthesized two novel NIR fluorescent probes 10 and 11 (Fig. 8). The two probes were highly sensitive to pH fluctuations (especially at the range of 5.50–4.00, with pKa values of 4.72 and 4.45) and could be reversibly turned off and on by alternating the pH values. The fluorescence imaging experiment on pH showed that the two probes can monitor the pH fluctuations of living cells because of their good membrane permeability. The two probes show special potential in detecting pH fluctuations in biological systems [25].

**Fig. 9:**
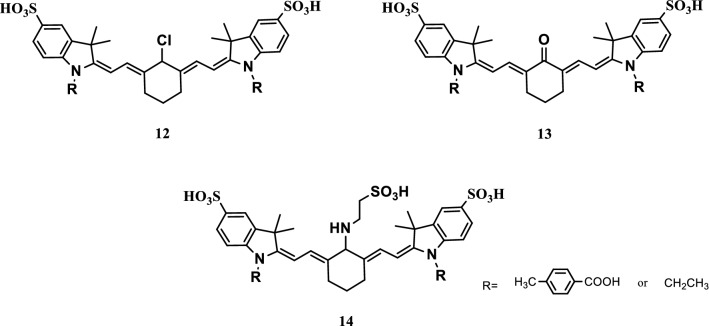
Structures of probes 12, 13, and 14

Six N-p-carboxybenzyl(or ethyl)-5,5′-bisulfonic heptamethine cyanines were developed to detect extreme pH values in biological systems, wastewater, and/or sol/gel formation. They exhibited different pH responses in water, ethanol, and/or methanol under extreme pH conditions. The structure of SCy-Os (Fig. 9. probe 12, pKa of 3.02–3.09) reversibly changed into a tautomorphic isomer (SCy-OHs) at pH 2.11–4.28. Its maximum absorption and emission wavelength changed from 504–639 nm to 709–762 nm, and the color of the solution changed from red to green. At the same time, the corresponding strength of the solution immensely was changed. SCy-Cls and SCy-Ns were tautomerized under strong alkaline conditions (pH 9.80–13.90, pKa of 10.41–11.93) because of the difference between the intermediate atoms (Cl, N, O) of the cyanine molecules and the conjugated system. The cyanine molecules in the aqueous solution interacted with CTAB to change the absorption, emission wavelength, and intensity of cyanide. The pH responses changed under strong acidic conditions, and the CMC of CTAB significantly reduced. The interaction of cyanine with SiO_2_ sol and titanium dioxide sol particles affected the pH responses. Therefore, the probe was applicable to the manufacturing of particular materials because of their special sensitive pH responses under extremely complex acidic and alkaline environments [26].

**Fig. 10:**
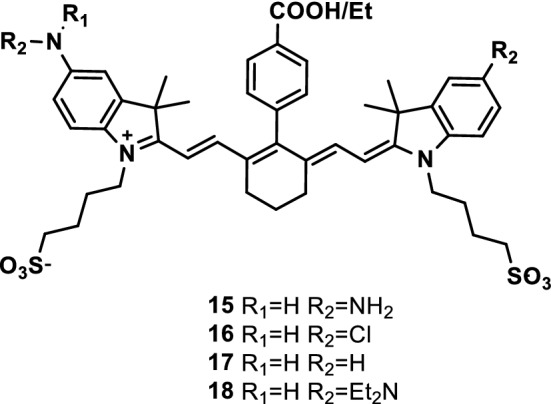
Structures of probes 15–18

Zhang et al. [27] developed four NIR fluorescence probes to show that pH in physiological environments could switch NIR fluorescence and photothermal efficiency. Probe 16 (Fig. 10, pKa_fluo_ 4.6) maximized its NIR fluorescence intensity in the acidic lysosome cavity and the photothermal effect in the alkaline mitochondrial matrix, specifically visualizing and removing various cancer cells, by fine-tuning the pKa values of these probes. This probe could facilitate image-guided tumor ablation by completely eradicating the tumors caused by organelle acidity and basicity disorder and improving prognosis.

**Fig. 11:**
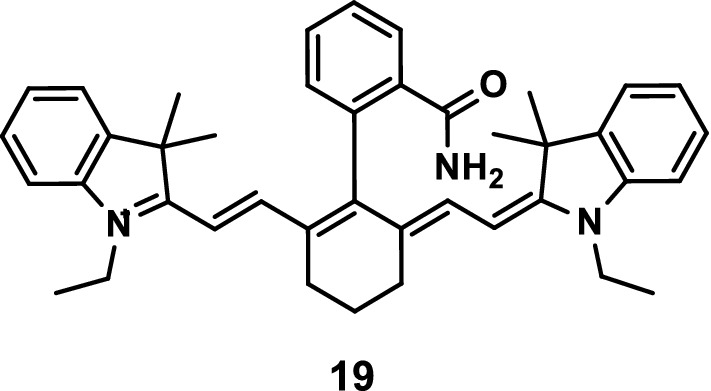
Structure of probe 19

He et al. [28] developed the NIR fluorescence probe 19 (Fig. 11) to prove that the internal fluorescence switch by spirocyclization in cyanines could be effectively used in the development of NIR pH detectors. They indicated that the probe 19 could be used to monitor the changes in pH values in biological systems in real time.

**Fig. 12:**
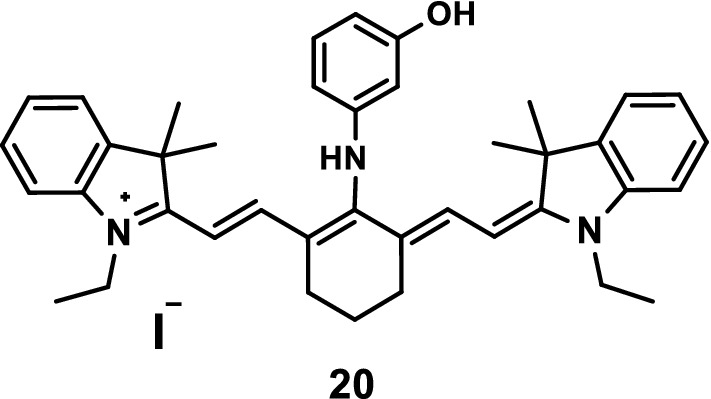
Structure of probe 20

A new NIR fluorescence probe 20 (Fig. 12) was designed and synthesized by introducing 3-aminophenol into the parent nucleus of indole heptamethine cyanine dye. A light stability experiment showed that probe 20 had good light stability. Phagocytic experiments showed that the probe had good cell membrane penetration and strong intracellular pH sensitivity. The pH titrations indicated a more than tenfold increase in fluorescence intensity within the pH range of 4.0–6.5 with a pKa value of 5.14, which was valuable for studying acidic organelles in living cells, and a pKa value of 11.31 within the pH range of 10.5–11.8. The fluorescence imaging of HepG2 cells showed that the probe could monitor the changes in hydrogen ion concentrations in living cells [29].

**Fig. 13:**
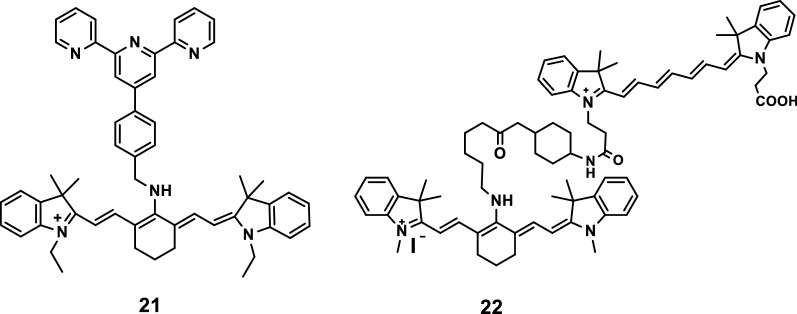
Structures of probes 21 and 22

Tang Bo’s experimental group [30] designed and synthesized a new pH-sensitive probe 21 (Fig. 13) to monitor slight fluctuations in pH values. The probe showed strong sensitivity with changing in hydrogen ion concentrations at the pH range of 6.70–7.90. The experimental results showed that the probe performed real-time in situ fluorescence imaging in living cells, effectively avoided the interference of biological tissues and spontaneous fluorescence, and exhibited important biological application value. In the same year, Kiyose et al. [31] introduced a NIR pH fluorescence probe on the basis of the FRET mechanism. The structure of probe 22 is shown in Fig. 13. The absorption and emission peaks of the probe red shifted when it was combined with H^+^.

**Fig. 14:**
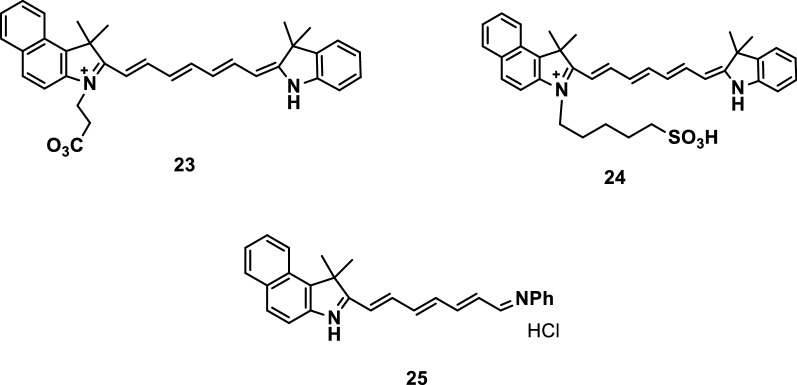
Probes 23–25 with pH-sensitive activities

Zhang et al. [32] synthesized three NIR fluorescence probes 23, 24, and 25 with pH-sensitive activities, as shown in Fig. 14. The removal of an N-substituted side chain in the ICG molecule by the three probes resulted in their pH sensitive characteristics in the NIR and related physiological range.

**Fig. 15:**
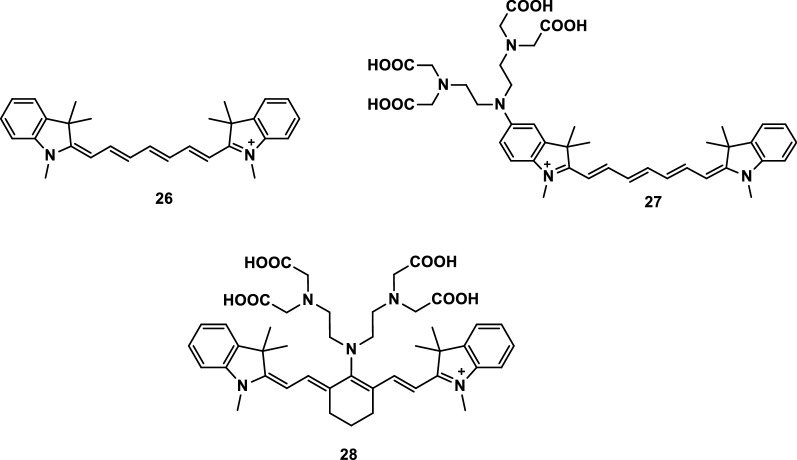
Probes 26–28 with pH-sensitive activities

Mikhail et al. [33] developed three probes (Fig. 15) with pH-sensitive activities. The difference in the pH sensitivities of the three probes were mainly due to the electron coupling through the tertiary amine in their molecular structure and the dependence of the pKa sensitivity of the probes on the location of the amine. All three probes can be used to detect pH activity.

### Application of metal ion selective NIR Fluorescence probes

The body contains a small amount of various metal ions with important physiological functions. Mg^2+^ is a metal ion with the most extensive life activities in the body [34] and is involved in various physiological functions, such as extensive material metabolism and energy metabolism. Zn^2+^ is widely involved in cell growth, neural transmission, enzyme catalysis, and other physiological functions [35, 36]. It can also maintain the normal metabolic function of Vitamin A and maintain the growth and development of the body. In addition, Zn^2+^ is a cofactor or activator of many enzymes. [37] Cu^2+^ is related to the regulation of melanin formation, hemoglobin production, and elastic tissue structure in vivo [38]. Studies have shown that the disorder of metal ions in the body will lead to the disorder of the physiological functions of the body and easily cause the formation of various diseases [39–41]. Therefore, a series of metal ion detection probes in vivo and in vitro should be developed.

**Fig. 16:**
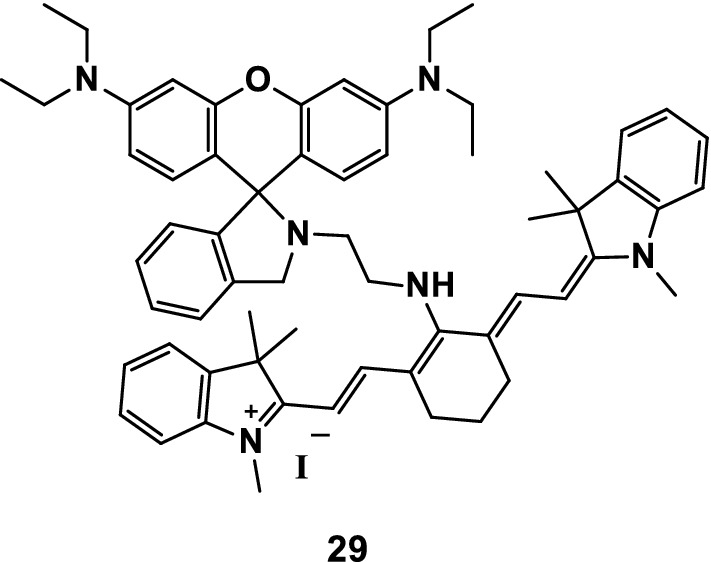
Structure of probe 29

Li et al. [42] reported the probe 29 (Fig. 16) that was used to detect Fe^3+^ and Cu^2+^ in MeOH/H_2_O and MeCN/H_2_O solvents, respectively. The detection limits of Fe^3+^ and Cu^2+^ detected by the probe were 0.737 μM and 1.019 μM, respectively. Compared with other common coexisting metal ion probes, probe 29 had stronger selectivity and higher sensitivity when detecting Fe^3+^ and Cu^2+^. The fluorescence imaging of Cu^2+^ in SH-SY5Y109 cells in vivo proved that the probe offered practical value in biological systems.

**Fig. 17:**
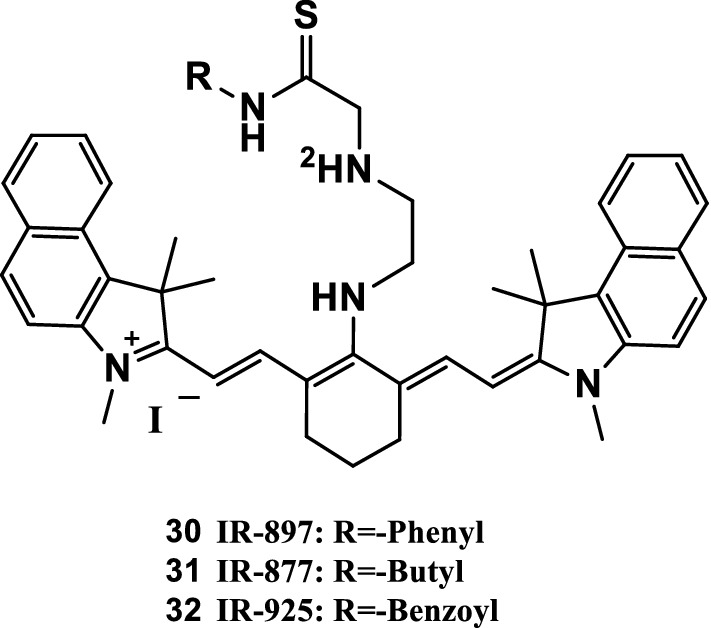
Structure of probes 30–32

Three tricarbocyanine dyes (Fig. 17, IR-897, IR-877, and IR-925) with different thiourea substituents were used as dosimeter units through specific Hg^2+^-induced desulfurization. These dyes were evaluated using a fast indicator paper for Hg^2+^ and MeHg^+^ ions. Compared with existing Hg^2+^ selective chemical dosimeters, IR-897 and IR-877 had more convenient synthesis, longer wavelength, and higher molar extinction coefficient in the NIR, and less interference to Ag^+^ and Cu^2+^. The red shifts of the three dyes were apparent, resulting in a clear change in color from dark blue to bean-green, and the red shifts could be used as a useful indicator. Additionally, experiments with living SW1116 cells showed that these three tricarbocyanine dyes with low toxicity could exhibit special characteristics that were favorable for visualizing intracellular Hg^2+^ and MeHg^+^ ions in biological systems, including excellent membrane permeability, minimal interfering absorption and fluorescence from biological samples, low scattering, and deep penetration into tissues. [43].

**Fig. 18:**
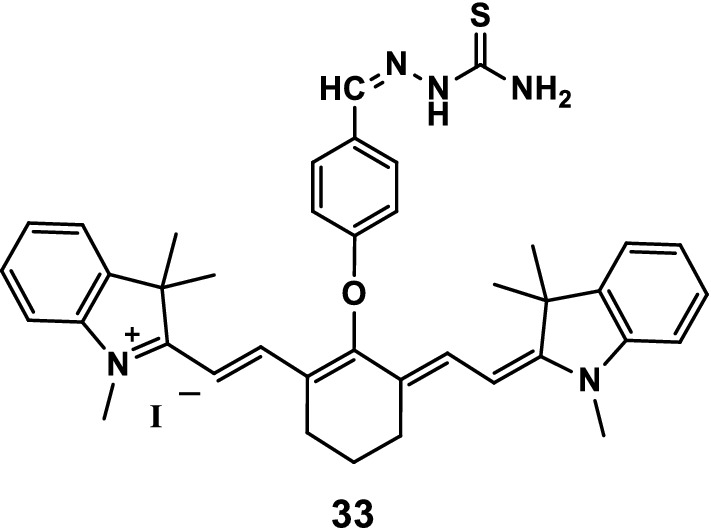
Structure of probe 33

Gao et al. [44] reported a novel type of NIR heptamethine cyanine ligand that selectively binds Hg^2+^ through the polymerization of monomers of NIR ligands. The sensor was simple and effective, and its detection limit in aqueous solution was 1.93 × 10^−8^ M. It had high sensitivity because of its unique sensing mechanism. Thus, the recognition of a small amount of Hg^2+^ led to the aggregation of probe 33 (Fig. 18), resulting in a change in the absorption spectrum. The results showed that probe 33 effectively detected Hg^2+^ and could thus serve as a good substitute for other sensors.

**Fig. 19:**
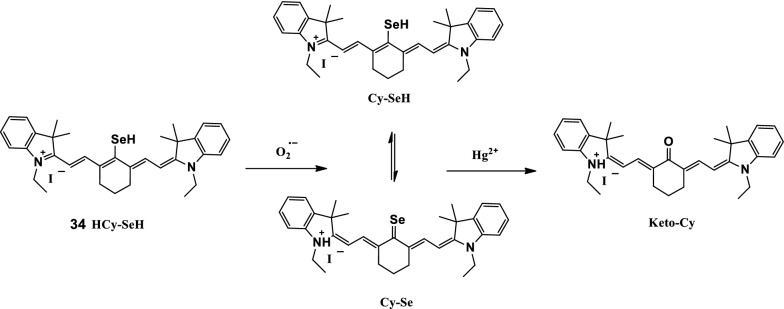
Molecular structure and the proposed mechanism for O_2_^·−^ and Hg^2+^ associated detection

Wang et al. [45] designed and synthesized a NIR three-channel fluorescent probe HCy-SeH (Fig. 19, probe 34) to detect O_2_^·−^and Hg^2+^ in chronic mercury poisoning models in cells and mice. During detection, the proportional fluorescence signal provided by the three-channel response eliminated the interference caused by uneven load or uniform distribution. The probe showed good selectivity and sensitivity for the associated detection of O_2_^·−^ and Hg^2+^. The probe was evaluated through fluorescence imaging and flow cytometry analysis for the in situ detection of O_2_^·−^ and Hg^2+^ in the HEK 293 cell model. The accumulation of Hg^2+^ destroyed the antioxidant system of cells and caused the overproduction of O_2_^·−^. The results showed that the probe could be a powerful tool for the association detection of O_2_^·−^ and Hg^2+^ in vitro and *vivo*.

**Fig. 20:**
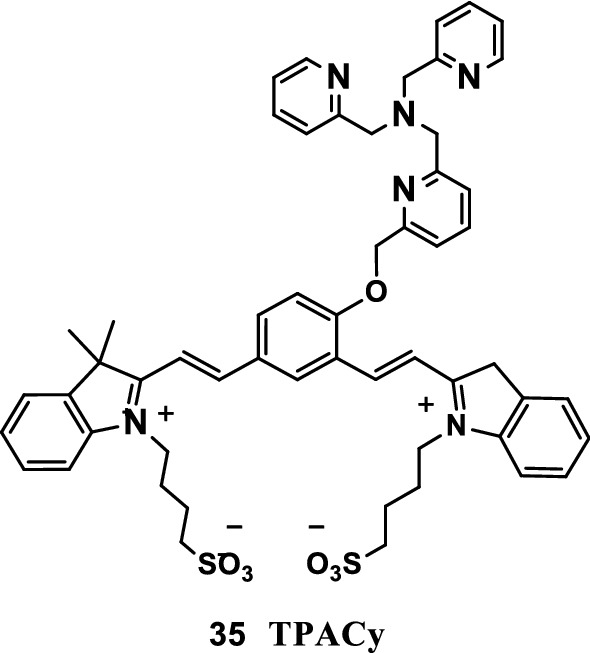
Structure of probe 35

Debabrat Maity’s team [46] successfully developed a Cu^+^ selective water-soluble switch “NIR fluorescence-ready” probe (TPACy, Fig. 20, probe 35). The probe easily reacted with Cu^+^ by releasing the NIR-emitting cyanine dye under the physiologically relevant pH range. The fluorescence dye of the probe released during the catalytic reaction of metal ions could be used to effectively detect the submicromolar concentrations of Cu^+^. The probe can be used as a noninvasive tool for detecting NIR fluorescence and the in vivo imaging of Cu^+^ pools in biological fluids.

**Fig. 21:**
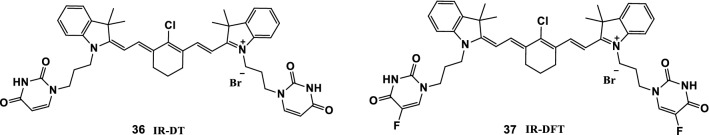
Structures of probes 36 and 37

**Fig. 22:**
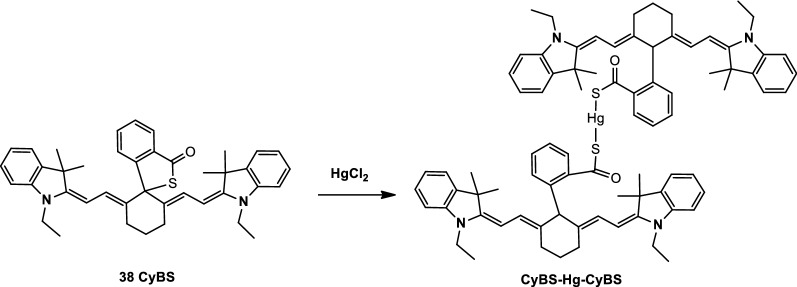
Possible sensing mechanism of 38 CyBS with Hg^2+^

Long et al. [47] synthesized two NIR fluorescence probes, IR-DT and IR-DFT (Fig. 21, probes 36, and 37), which contains two thymines on the N-sides. These dyes could form a T-Hg-T complex through thymine and bind specifically to Hg^2+^. IR-DFT had a better response to Hg^2+^ compare with IR-DT. It also had relatively high sensitivity and low detection limits for Hg^2+^ because fluorine-substituted thymine could accelerate self-aggregation and fluorescence quenching. The experimental results showed that Hg^2+^ in the mitochondrial region of the cells was successfully detected by IR-DFT. He et al. [28] also reported a NIR fluorescence probe CyBS (Fig. 22, probe 38) to detect Hg^2+^ in living cells and animals.

**Fig. 23:**
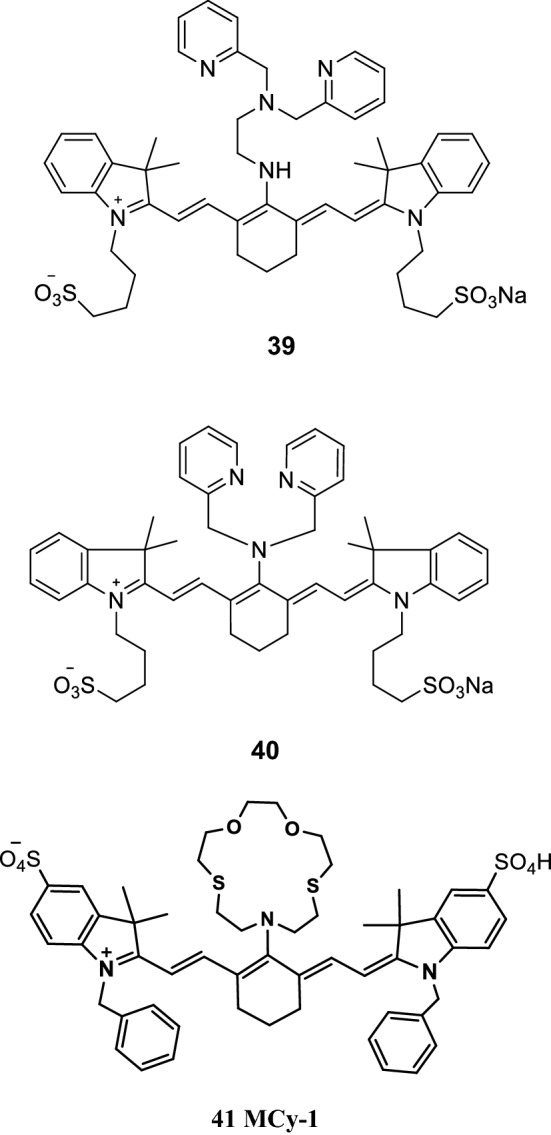
Structures of probes 39, 40, and 41

Kiyose et al. [48, 49] designed and synthesized a novel NIR fluorescence probe (probe 39, Fig. 23), with a maximum molar extinction coefficient of 7.0 × 10^4^ m^−1 ^cm^−1^ and large Stokes shift to detect Zn^2+^. The electron cloud density around dipyridine methyl ethylenediamine decreased, and the fluorescence signal of the probe increased when the recognition group of methyl amine in the probe bound to Zn^2+^. The results showed that the probe was suitable for the detection of Zn^2+^. Tang Bo’s team [50] reported a new NIR fluorescence probe (probe 40) for the detection of Zn^2+^ (Fig. 23). The recognition group of the probe was 2, 2-dimethyl-1-pyridine, and the fluorophore was indole heptamethine cyanine dyes. Fluorescence quenching occurs when the fluorescent probe was combined with Zn^2+^, and photoinduced electron transfer (PET) of the probe was inhibited.

Zhu et al. [51] reported a novel NIR fluorescence probe MCy-1 (Fig. 23, probe 41) to detect Hg^2+^ concentrations. The Hg^2+^ concentrations were obtained by introducing the recognition group of dioxane crown ether into the fluorescence group. The probe’s recognition group combined with Hg^2+^ affected the entire conjugate system of the macromolecule and changed the fluorescence signal. Thus, the presence of Hg^2+^ on the probe was observed by the naked eye. The results showed that the probe had good selectivity to Hg^2+^ and could thus easily detect the presence of Hg^2+^.

**Fig. 24:**
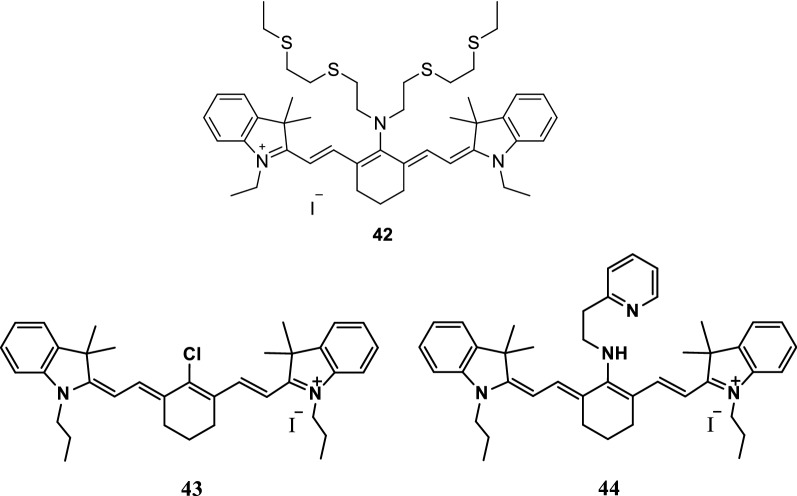
Structures of probes 42, 43 and 44

Cao et al. [52] presented an NIR fluorescence probe 42 (Fig. 24) with thioether ring as the recognition group and indole heptamethine cyanine dye as the fluorescent group to detect the presence of Cu^+^. Before the binding to Cu^+^, the recognition group provided electrons and weakened the fluorescence signal of the cyanine dyes. After the binding to Cu^+^, the fluorescence signal increased, and the absorption spectrum red shifted.

Han et al. [53] synthesized as a fluorescent probe 44 (Fig. 24) for detection of Cu^2+^ with high selectivity. The probe 44 was composed of 2-(2-Amino-ethyl) pyridine and IR-780 iodide (Fig. 24, probe 43), and the response of the probe was based on the fluorescence quenching upon binding to Cu^2+^. The sensing performance of the proposed Cu^2+^-sensitive the probe was then investigated. The probe can be applied to the quantification detection of Cu^2+^ with a linear concentration range covering from 4.8 × 10^−7^ to 1.6 × 10^−4^ mol/L and a detection limit of 9.3 × 10^−8^ mol/L. The experimental results showed that the response of the probe to Cu^2+^ was independent of pH in medium condition, and exhibited excellent selectivity towards Cu^2+^ over other common metal cations.

**Fig. 25:**
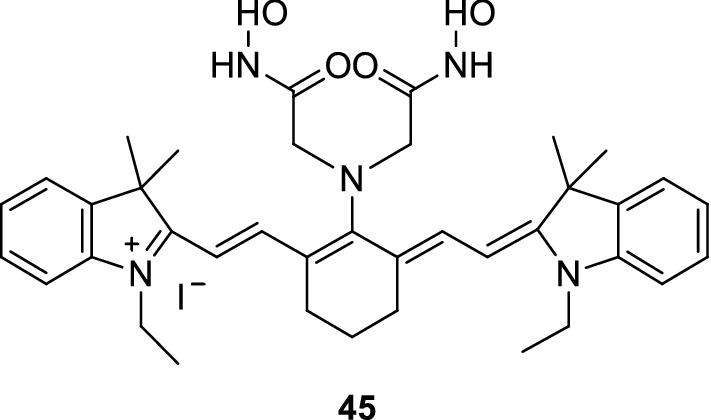
Structure of probe 45

Li et al. [54] synthesized a fluorescence probe 45 (Fig. 25) to detect Cu^2+^. The maximum absorption wavelength of the probe changed to blue when its recognition group bound to Cu^2+^. The increase in Cu^2+^ concentration boosted the fluorescence signal strength of the probe, thus increasing the fluorescence quantum yield to approximately six times. The experimental results showed that the probe had high affinity and selectivity for Cu^2+^ and recognized the presence of Cu^2+^.

**Fig. 26:**
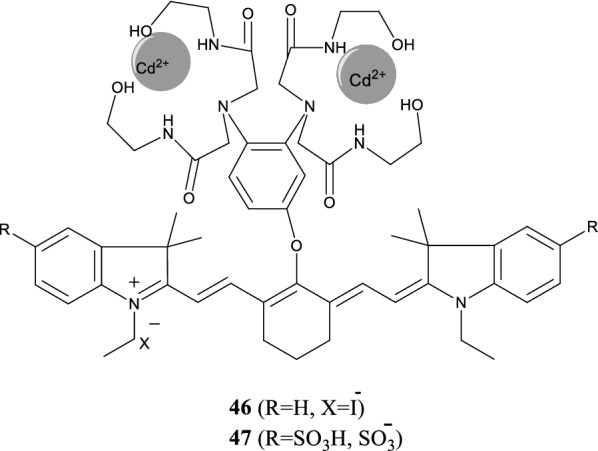
Structures of probes 46 and 47

**Fig. 27:**
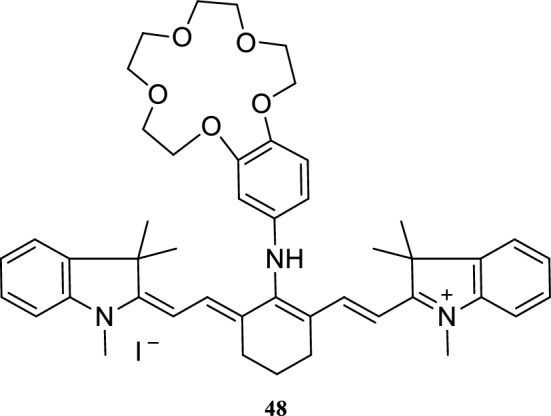
Structure of probe 48

Yang et al. [55] reported two NIR fluorescence probes 46 and 47 (Fig. 26) with good affinity and selectivity for Cd^2+^. Probe 47 has strong detection ability for Cd^2+^ and detected Cd^2+^ from a neutral solution and a solution mixed with other ions. Probe 48 (Fig. 27) consists of crown ether rings and indole heptamethine cyanine dye as the recognition and fluorescence groups, respectively. The experimental results showed that the probe could effectively identify and detect metallic lithium ions simultaneously [56].

**Fig. 28:**
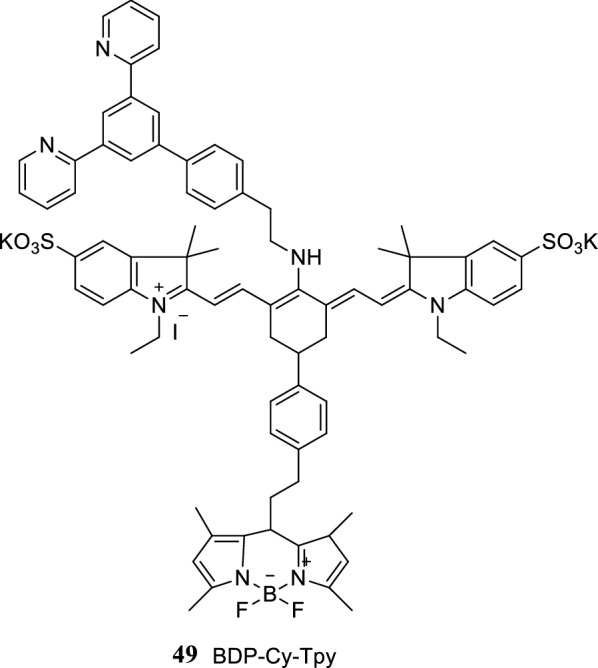
Structure of probe 49

Tang et al. [57] synthesized a probe 49 (BDP-Cy-Tpy) consisting of a fluorescent group of indole heptamethine cyanine dye and a recognition group of BODIPY to detect ferrous ions (Fig. 28). The probe emitted fluorescence signals when the ferrous ions combine with the recognition group of the probe. Fluorescence signals were observed at BODIPY, whereas no fluorescence signal was observed in the indole heptamethine cyanine dye with the combination of ferrous ions and the recognition group of the probe. Therefore, the probe is effective in detecting the presence of ferrous ions.

## Application of small molecule-sensitive NIR fluorescence probes

### Fluorescent probes to detect active sulfur

**Fig. 29:**
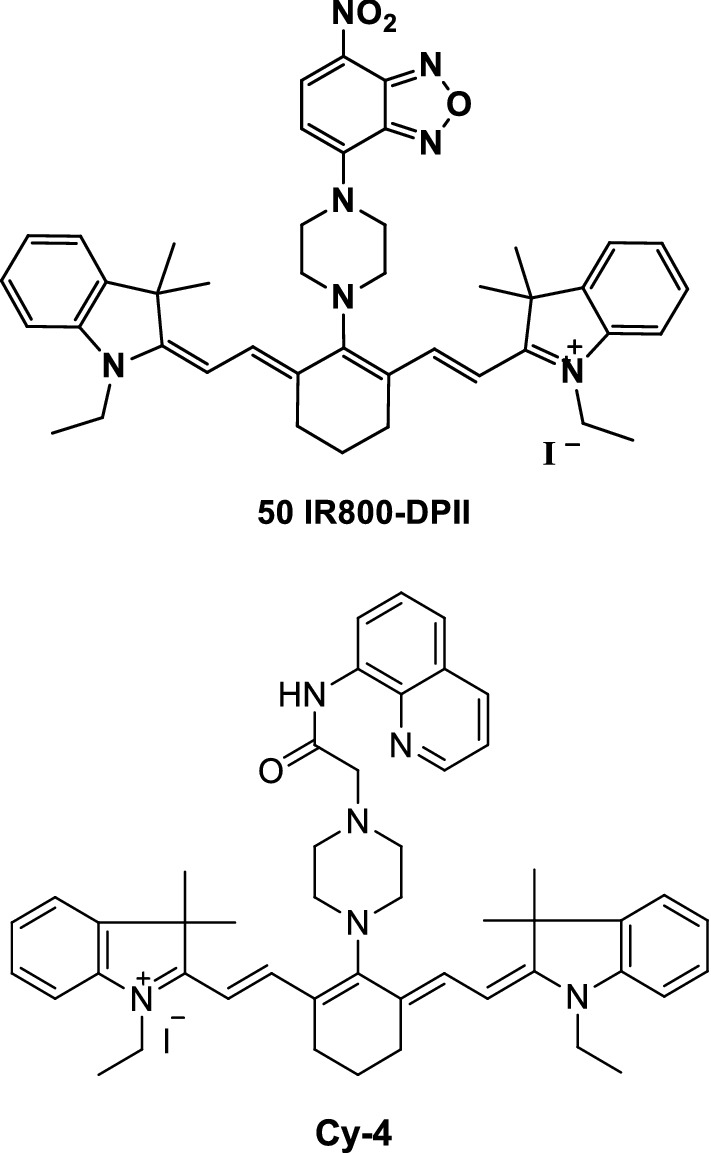
Structures of probes 50 and Cy-4

Sulfides play a unique role in biological systems, and the development of many sensitive probes is crucial for the fluorescence detection of S^2−^. An NIR fluorescence probe IR800-DPII (Fig. 29, probe 50) was synthesized by heptamethine cyanine and NBD as an “off–on” fluorescence sensor for detecting S^2−^ using the matrix-induced cleavage mechanism. Its detection limit for S^2−^ on neutral particles is 0.15 M, which indicates better selectiveness than other biologically related analytes. The probe is successfully applied to living cells, and the fluorescence imaging of S^2−^ in living cells is achieved. The principle of group removal from the probe is the response induction of S^2−^ by conducting a closed attack on the NBD group of the probe. The probe is suitable for measuring the accumulation of S^2−^ for a time period because of the slow cleavage reaction [58].

Cao et al. [59] synthesized an NIR fluorescence probe Cy-4 (Fig. 29) to detect the presence of sulfur ions. The probe exhibited fluorescence signals under normal conditions. However, the fluorescence signals were quenched when the nitrogen atom of the identification group bound to copper chloride. As the binding ability of sulfur ion to copper was stronger than that of nitrogen atom, the solution containing sulfur ion was added to the solution after fluorescence quenching. Moreover, the complexed copper ion combined with sulfur ion changed the molecular structure of the probe into its original state to generate fluorescence signals.

**Fig. 30:**
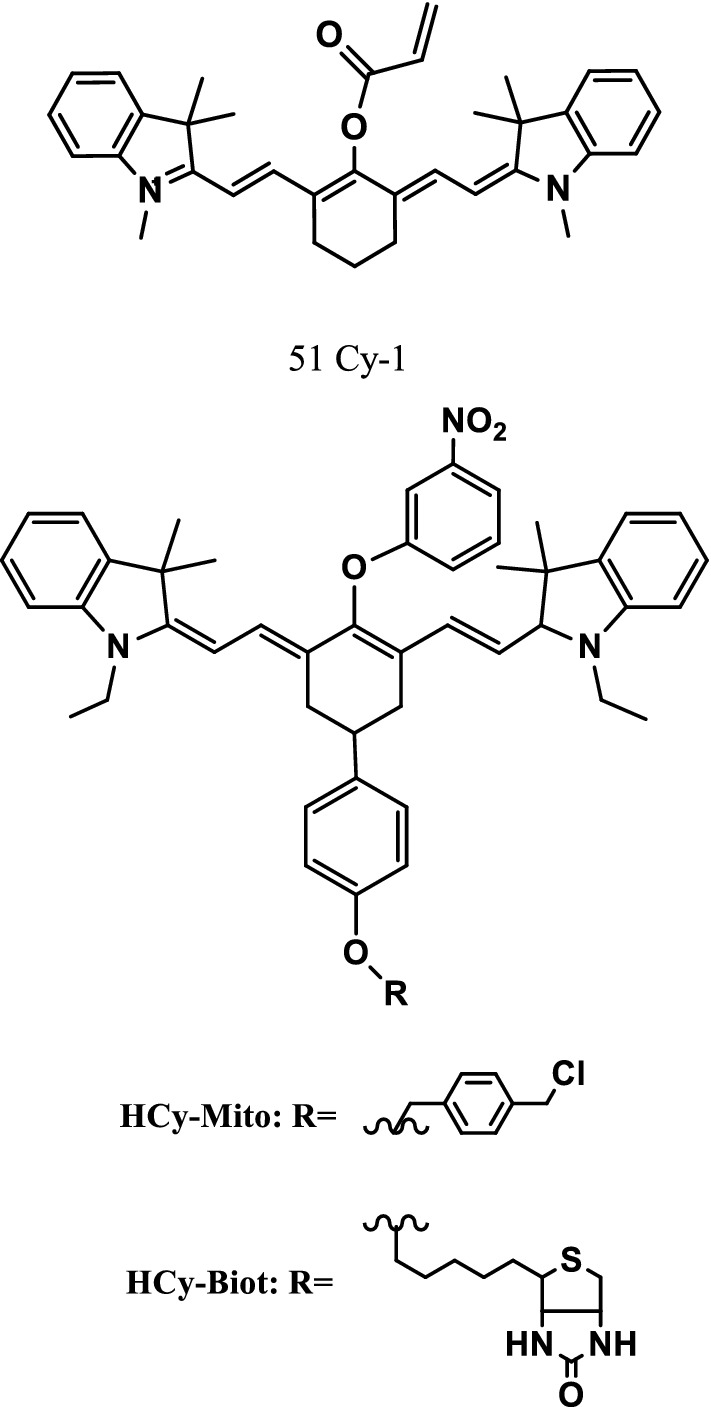
Structures of probes 51, HCy-Mito, and Hcy-Biot

A novel NIR fluorescence probe Cy-1 (Fig. 30, probe 51) was presented to detect hydrosulfide. The reaction mechanism of the probe was a nucleophilic reaction in which HS^−^ replaces acryloyl moiety. Cy-1 exhibited a rapid fluorescence quenching (700 nm excitation) and a fast fluorescence enhancement (544 nm excitation) with the addition of NaHS. Its absorption spectra and colors were clearly observed by the naked eye in tens of seconds. Cy-1 had better selectivity to Cys compared with CyAc. The probe showed excellent selectivity and sensitivity to HS^−^ on various anions (0.33 μM detection limit). The probe was successfully used in the imaging of hydrogen sulfide (H_2_S) in fetal bovine serum samples and living cells [60].

Recent studies showed that hydrogen polysulfide (H_2_S_n_) was a true intracellular signaling molecule that played an important role in the cardiovascular and nervous systems [61]. Fabiao Yu et al. [62] reasoned that the balance of O_2_^·−^ and H_2_S concentrations in vivo played a key role in human physiological and pathological processes, so they developed two NIR fluorescence probes, Hcy-Mito and Hcy-Biot (Fig. 30), for the detection of O_2_^·−^ and H_2_S in vivo. Studies showed that Hcy-Mito was highly sensitive and selective to endogenous O_2_^·−^ and H_2_S_n_. Flow cytometry assays for apoptosis confirmed that H_2_S_n_ played an important role in the antioxidant system, and the assay results of RAW264.7 cells and HUVECs showed that H_2_S_n_ might be caused by mitochondrial oxidative stress. H_2_S_n_ could protect cells from mitochondrial oxidative stress by directly removing O_2_^·−^. In vivo near infrared fluorescence imaging experiments showed that the probe had a strong penetration depth and could detect O_2_^·−^ and H_2_S_n_ in vivo.

### Fluorescent probes to detect reactive oxygen species (ROS)

Under the normal physiological metabolism of the body, hydrogen peroxide (H_2_O_2_), superoxide anion radical (O_2_^·−^), hydroxyl radical (HO·), peroxynitrite (ONOO^−^), and other reactive oxygen radicals are produced and perform important functions in the body. The increase in the level of intracellular oxidation can cause abnormalities in biological molecules and the development of some diseases, such as cancer, tissue peroxidation, and inflammation. At present, the commonly used methods for the determination of ROS are electron paramagnetic resonance, chemiluminescence, and high-performance liquid chromatography. Few methods are available to detect free radicals in vivo because of the short half-life of ROS. NIR fluorescence imaging can detect ROS or tissue fluorescence in cells because of its unique fluorescence characteristics [63]. Therefore, fluorescent probes with high sensitivity, low cost, minimal errors, and strong specificity should be developed for the detection of ROS.

**Fig. 31:**
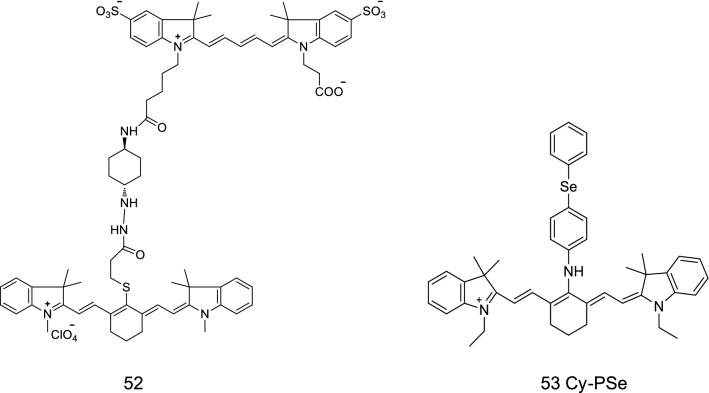
Structures of probes 52 and 53

A NIR fluorescence probe 52 (Fig. 31) was presented by Oushiki et al. [64] The probe was formed through the covalent combination of two cyanine dyes, pentamethylcyanine and heptamethylcyanine. The reactivity of the two cyanine dyes was relatively different from that of reactive oxygen radicals. The probe could detect ROS free radicals and use for imaging in mice with peritonitis.

Yu et al. [65] designed and synthesized a new type of NIR fluorescence probe (Fig. 31, probe 53) to detect the presence of peroxynitrite ions. The probe did not react with ROS. The fluorescence quenching of the fluorescent probe was caused by the PET effect of selenium on the entire methionine chain. The PET function of the Cy-P_Se_ probe was inhibited by oxidation with the addition of oxygen nitrite ions, resulting in the production of a fluorescence signal. The oxidized selenium reduced and returned to the molecular structure of the probe itself under the action of the enzymatic catalytic cycle. Hence, the probe is suitable for many biological applications.

**Fig. 32:**
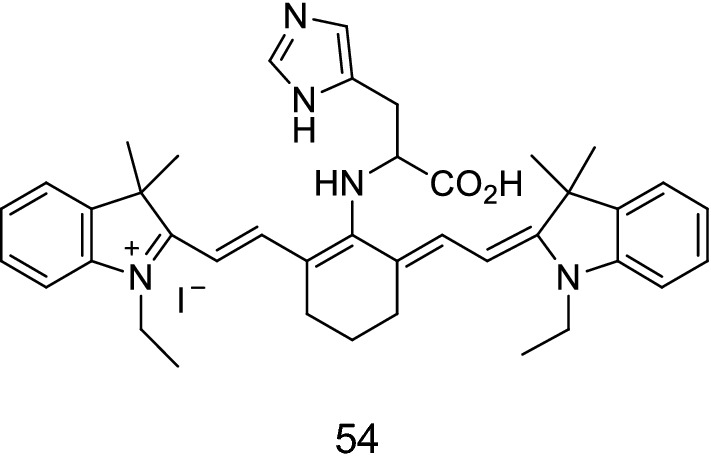
Structure of probe 54

Xu et al. [66] reported an indole heptamethine cyanine probe 54 with high sensitivity and selectivity to detect singlet oxygen in the mitochondria (Fig. 32). The probe resisted the interference of biological systems, displayed the singlet oxygen of cells, and had low cytotoxicity and strong stability. Experiments showed that the probe could be used to detect ^1^O_2_ in cells in vivo.

**Fig. 33:**
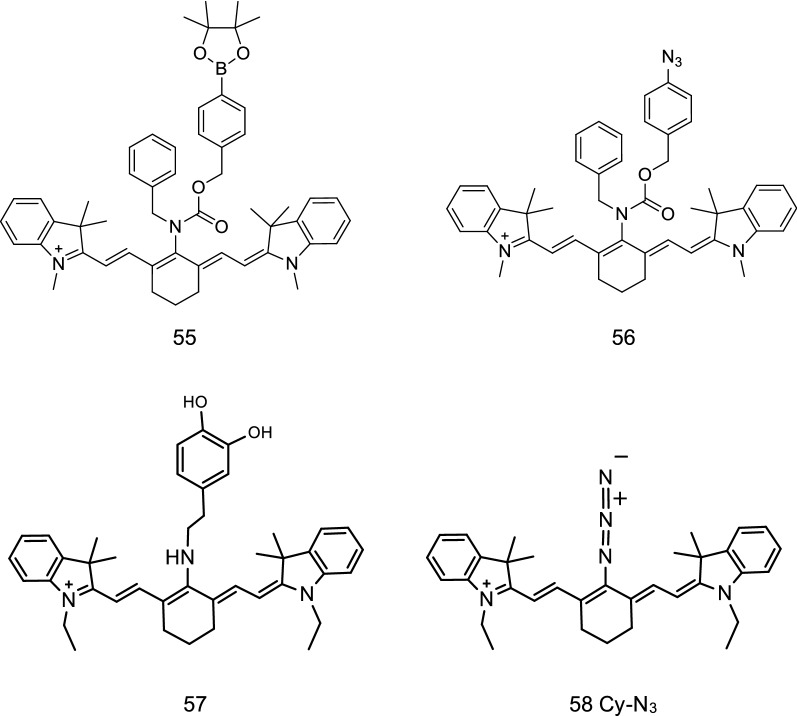
Structures of probes 55, 56, 57, and 58

H_2_O_2_ and H_2_S are small molecule signal sensors in vivo that play an important role in the pathological and physiological processes of organisms. In 2012, Wang et al. [67] reported an NIR fluorescence probe to detect the reduction of H_2_S in cells using cyanine dyes as fluorescence agents and a nitro group as the functional group. Two fluorescence probes 55 and 56 were synthesized by Zhu et al. [68] to detect small molecules (Fig. 33) through the change of fluorescence ratio. The probes were found to be effective in detecting the presence of H_2_S and H_2_O_2_ in the body. During the experiment, no significant change was observed in the fluorescence intensity of probes 55 and 56 under the presence of other ROS or active nitrogen in the solution. Probe 55 showed a high degree of selectivity to H_2_O_2_ under the presence of H_2_O_2_ in the solution. The fluorescence intensity of probe 56 was significantly enhanced with the presence of H_2_S in the solution, indicating its high selectivity to H_2_S. The fluorescence stability experiment showed that the two probes had stable fluorescence characteristics in the range of the physiological pH. Confocal imaging experiments confirmed that the probes could be used to detect the presence of H_2_O_2_ and H_2_S molecules in vivo.

Yu et al. [69] synthesized an NIR fluorescence probe DA-Cy for the detection of H_2_O_2_ (Fig. 33, probe 57). The probe used catechol as the identification group and indole heptamethine cyanine dye as the fluorophore. Yu et al. [70] developed an NIR fluorescence probe Cy-N_3_ (Fig. 33, probe 58) for the detection of H_2_S in chemical substances. The probe molecule used azide as the identification group and cyanine dye as the fluorophore. The azide group became an electron group with the binding of oxygen sulfide to the amino group, resulting in the change of spectral energy before and after the reaction.

**Fig. 34:**
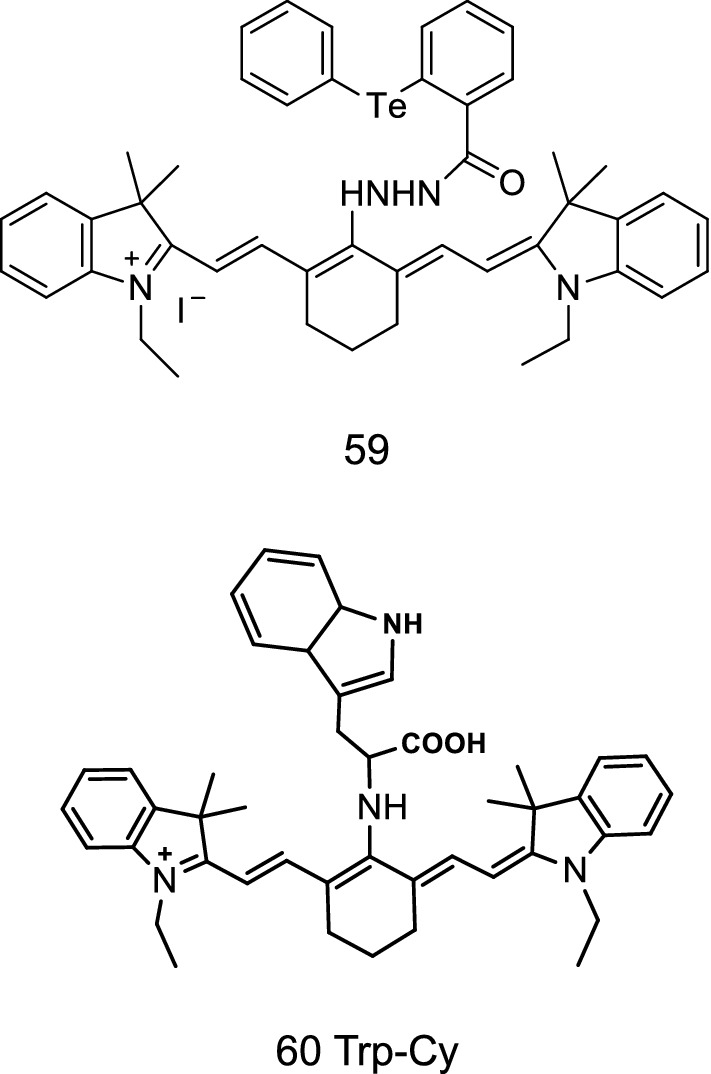
Structures of probes 59 and 60

ONOO^−^ is a kind of oxidant in the human body, and its instability may lead to inflammation, neurodegenerative diseases, and other conditions. ONOO^−^ can greatly affect the content of GSH in body cells and thereby cause damage to the body. Therefore, the redox state between ONOO^−^ and GSH should be detected in vivo. Yu et al. [71] synthesized the probe 59 (Fig. 34) to detect the balance of ONOO^−^/GSH in vivo. The fluorescence signal of the probe was enhanced and saturated in a short time period when it was combined with ONOO^−^, and the probe had high selectivity to ONOO^−^. The probe could be used to detect ONOO^−^ in cells with low cytotoxicity in cell experiments.

Xu et al. [72] designed and synthesized an indole heptamethine cyanine probe Trp-Cy (Fig. 34, probe 60) to detect ozone. The probe molecule is a tryptophan recognition group. Tryptophan had a distorted intramolecular charge transfer effect on the cyanine dyes, causing the blue shift of the maximum absorption peak. The structure of the indole ring was destroyed, and the twisted charge effect disappeared with the addition of ozone. This condition caused the reaction product molecule to undergo two wavelength red shifts. The probe could detect ozone in living cells.

**Fig. 35:**
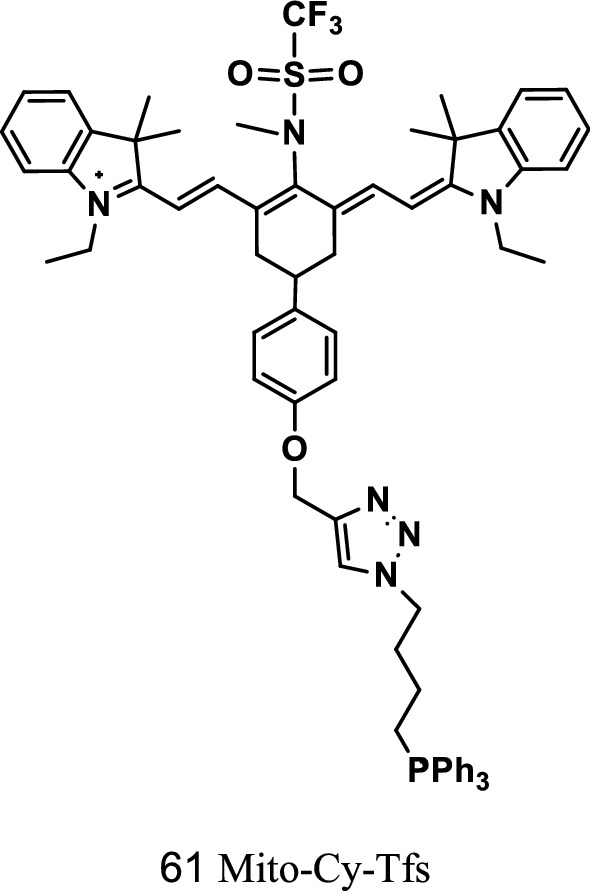
Structure of probe 61

Han et al. synthesized a rate-based NIR mitochondrial targeting fluorescent probe Mito-Cy-Tfs (Fig. 35, probe 61) to detect the changes in the level of O_2_^·−^ in cells and to assess the relationship of O_2_^·−^ concentration and apoptosis degree in I/R. The probe displayed deep tissue permeation for real-time imaging of O_2_^·−^ concentration in the liver of I/R mice. The experiment proved that the probe could be used to indicate and evaluate the correlation of O_2_^·−^ level and the degree of organ damage in I/R, IPC, and IPTC processes [73].

**Fig. 36:**
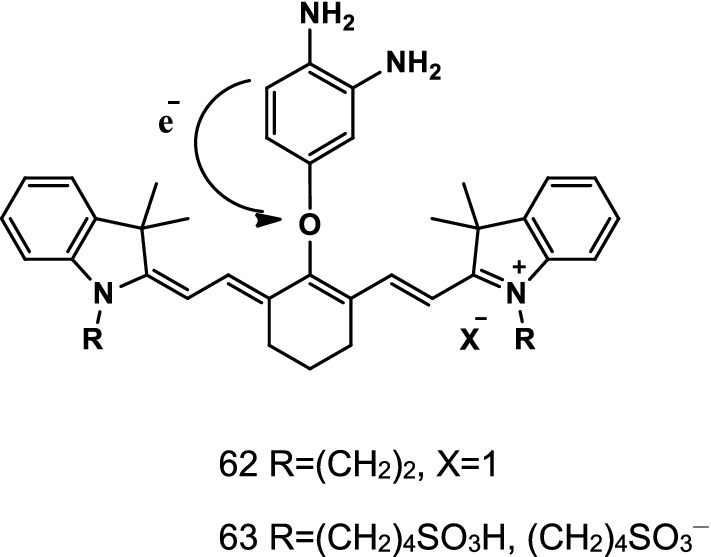
Structure and photochemical mechanism of NO fluorescent probe

Sasaki et al. [74] synthesized two NIR fluorescence probes 62 and 63 for the determination of nitric oxide (NO), as shown in Fig. 36. The fluorescence penetration of the probe increased, and the background interference of spontaneous fluorescence was minimal with the small light absorption of the body. The fluorescent probes were suitable for the detection of NO in vivo. In the presence of NO, an enhanced fluorescence signal was observed in the probe molecule because of PET. In vivo imaging experiments confirmed that these probes could be used to detect the presence of NO in biological organs.

### Fluorescent probes to detect glutathione and/or cysteine

**Fig. 37:**
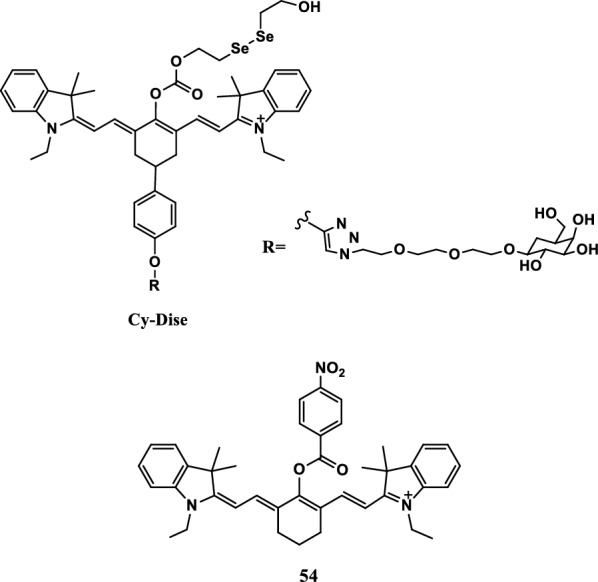
Structures of probes Cy-Dise and 54

Glutathione (GSH) is the most abundant non-protein biothiol in cells, which can protect and prevent cell apoptosis. Yu et al. [75] successfully synthesized a ratiometric fluorescent probe (CyO-Dise, Fig. 37) based on a selenium–sulfur exchange reaction to detect of GSH concentration fluctuations in vivo and in vitro. The probe had been successfully used to detect changes in GSH concentration in HepG2 and HL-7702 cells under low temperature and hyperthermia. CyO-Dise was composed of fluorescent cyanine, bis (2-hydroxyethyl) dienoamide and d-galactose, and the probe could detect the presence of GSH within 35 s in the selenium-sulfur exchange reaction. In addition, the probe could be used to image the GSH levels of HepG2 and HepG2/DDP xenografts in vivo.

Yin et al. [76] synthesized a NIR colorimetric fluorescence probe Cy-NB (Fig. 37, probe 54) to selectively detect GSH and cysteine (Cys) in the mitochondria for indicating oxidative stress. Indole heptamethine cyanine dye was used as the fluorescent group of Cy-NB, and p-nitrobenzoyl was used as the fluorescent regulator. The uncaged p-nitrobenzoyl rearranged the polymethine π-electron system of the fluorophore with the triggering of Cys, leading to remarkable spectrum shifts in absorption and emission profiles. A quantitative fluorescence signal of Cys with a detection limit of 0.2 μM within 5 min was obtained on the basis of the aforementioned spectral characteristics. The Cy-NB probe sensitively detected the changes of the mitochondrial Cys pool in the HepG2 cells under different oxidative stresses. Cy-NB was successfully used in the imaging of Cys level changes in live mice. Mitochondrial Cys could be used as a biomarker of toxic stress and utilized in clinical applications.

**Fig. 38:**
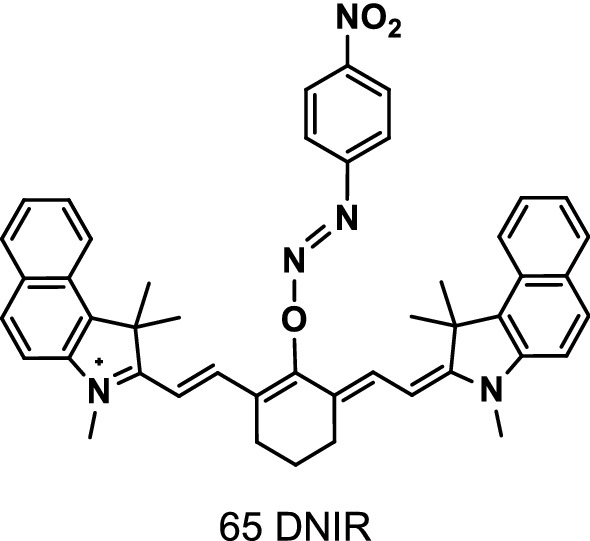
Structure of probe 65

Liu et al. [77] reported a heptamethine cyanine probe DNIR (Fig. 38, probe 65) to detect GSH and its oxidative form (GSSG) in blood. The NIR excitation wavelength was 804 nm, and the NIR emission wavelength was 832 nm. The probe contained a fluorescent group of heptamethylene cyanine linked to the functional group of nitro azoaryl ether. It had rapid reaction (3 min) and good selectivity toward GSH. The selectivity of the probe to GSH was better than that to GSSG and other amino acids (AAs). The probe rapidly reacted to GSH, especially in the presence of GSH reductase and nicotinamide dinucleotide phosphate, and showed good performance for the indirect detection of GSH without requiring separation prior to fluorescence measurement.

### Fluorescent probes to detect selenocysteine

**Fig. 39:**
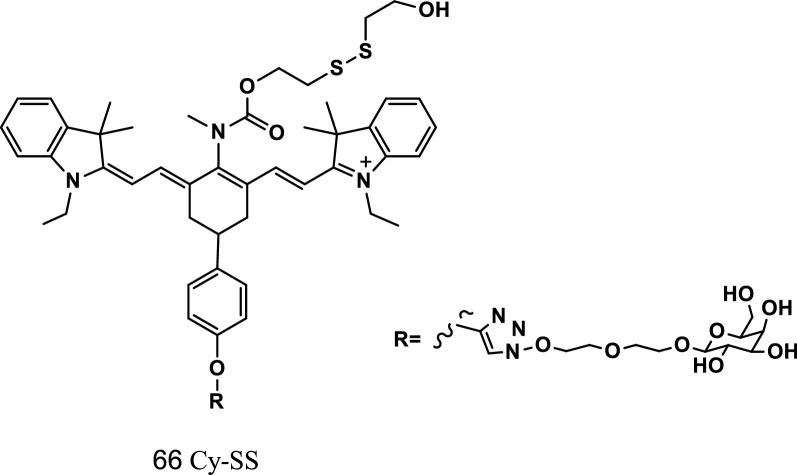
Structure of probe 66

Selenocysteine (Sec) is a highly reactive and unstable active selenium in cells. Although it has antioxidant activity in various liver diseases, Sec in living cells is difficult to determine in vivo. Han et al. reported a proportional NIR fluorescence probe Cy-SS (Fig. 39, probe 66) consisting of a heptamethylcyanine fluorescent group, reaction unit bis (2-hydroxyethyl) disulfide, and liver targeting d-galactose to quantitatively and qualitatively measure Sec in living cells in vivo. On the basis of the detection mechanism of the selenium-sulfur exchange reaction, the Sec concentrations in HepG2, HL-7702 cells, and primary mouse hepatocytes were 3.08 ± 0.11 × 10^−6^, 4.03 ± 0.16 × 10^−6^, and 4.34 ± 0.30 × 10^−6^ m, respectively. The experiment showed that the probe had high selectivity in the liver. The specific fluorescence signal of the probe could be used to quantitatively analyze the changes of Sec concentrations in cells and acute hepatitis mice models [78].

### Fluorescent probes to detect cyanide anion

**Fig. 40:**
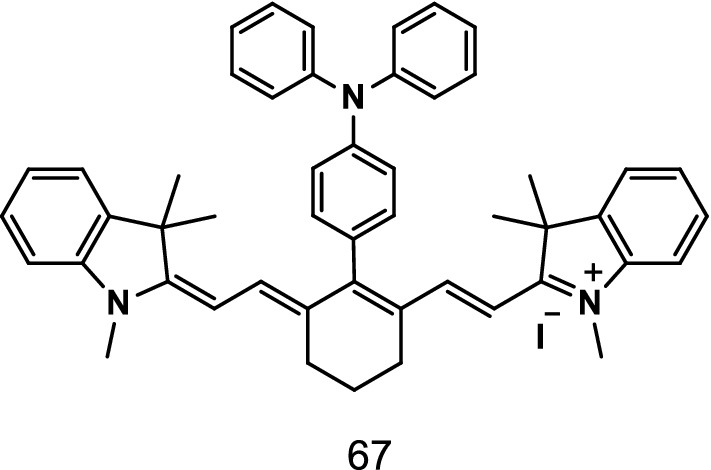
Structure of probe 67

Liu et al. reported a NIR fluorescence probe T-Cy (Fig. 40, probe 67) to detect CN^−^. The ultraviolet–visible (UV–vis) absorption and fluorescence spectra of the probe significantly changed with the addition of CN^−^, and the color changes were visible to the naked eye. The change in the spectral signal was linearly proportional to the CN^−^ concentration. The detection limits of the probe were 14 nM and 0.23 μM in CH_3_CN and CH_3_CN/H_2_O (9/1, v/v), respectively. The probe showed high selectivity and anti-interference to CN^−^ under the presence of other common anions (F^−^, AcO^−^, Br^−^, NO_2_^−^, Cl^−^, SO_4_^2−^, I^−^, HCO_3_^−^, CO_3_^2−^, SCN^−^) and biothiol (Cys/HCy/GSH). The test strip of the probe was suitable for detecting CN^−^, and fluorescence imaging showed the potential biological value of the probe [79].

Chen et al. [80] synthesized a detection of cyanide ion of NIR fluorescence probe Cy-I (Fig. 24, probe 44), and probe the Cy-I identify groups with copper ions, since light induced effects of electron transfer fluorescence signal disappears, when after the above add cyanide ion in the solution, as a result of the combination of cyanide ion and copper ion ability was stronger, from cyanide ion probe Cy-I, PET effect change, fluorescence and recovery.
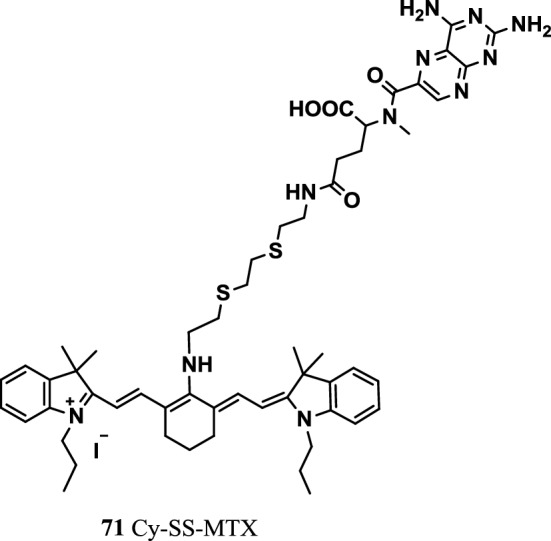


### Fluorescent probes to detect inorganic phosphate anions

**Fig. 41:**
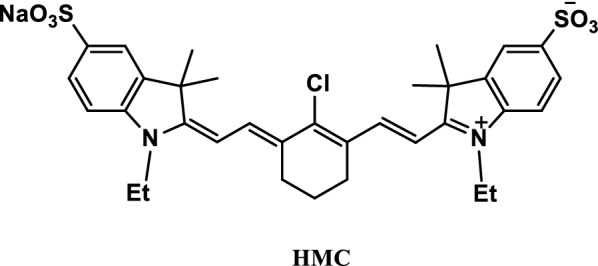
Structure of probe HMC

The absorbance of phosphate anion was determined using a NIR reagent. The method was tested with two reagents, namely, anionic heptamethine cyanine (Fig. 41, probe HMC) and Tb^3+^. At the specified concentration, the addition of Tb^3+^ reduced the maximum absorbance of heptamethine cyanine to 778 nm, but the absorbance returned to the original value with the addition of phosphate anion. Under optimal conditions, the reciprocal degree of absorbance was proportional to the concentration of phosphate anion. Other ions had small interference because phosphate anion had a special affinity to Tb^3+^. This method is successfully applied to determine inorganic phosphate anions in human saliva samples [81].

### Application of NIR fluorescence probes to labeled tumor cell types

Cancer is a general term for a large class of malignant tumors, and it poses a serious threat to human life and health. Millions of people die from cancer every year. Cancer has constantly been a top priority in life science and medical research [82]. Therefore, a low-cost, low-harm, real-time, and in situ method should be developed to detect the location, size, and status of tumors. The development of NIR fluorescence probes has turned this idea into a reality. [83].

Tumor and normal cells have different energy metabolism pathways. The glycolysis rate of malignant tumor cells is higher than that of normal cells, and normal cells mainly consume most glucose through the glycolysis pathway. The specific molecular mechanism of high glycolysis energy in tumor cells remains unclear. The high glycolysis activity of tumor cells may be related to the low expression of enzymes and transporters, high sensitivity of DNA to oxidative stress, and overexpression of glycolytic enzymes and glucose transporters.

**Fig. 42:**
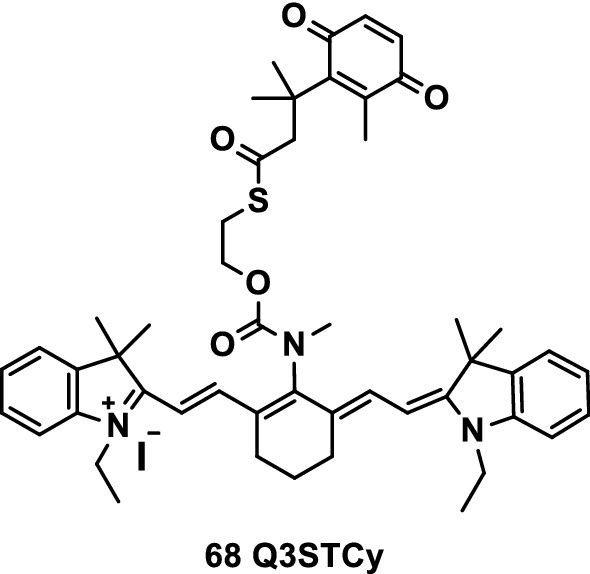
Structure of probe 68

Shen et al. [84] reported a novel NIR probe Q3STCy (Fig. 42, probe 68) to detect and visualize the NAD (P) H quinone reductase activity of endogenous cells in 2D and 3D cancer cell cultures and established preclinical in vivo models of diffused peritoneal ovarian cancer. hNQO1(quinone oxidoreductase isozyme I) specific reductive activation of the carefully crafted, electron-transfer quenched probe leaded to autonomous release of its corresponding tricarbocyanine reporter. The Q3STCy probe had an energy-specific NIR fluorescence emission that was stronger than that of inactive probes by several orders of magnitude. The probe/reporting system could detect and differentiate human cancer cells with different hNQO1 activity levels, including cells experiencing different microenvironments because of their location in multicellular tumor mimics. The probe dilution could be locally used in mouse xenotransplantation models to identify human ovarian cancer tumors smaller than 0.5 mm in size. Quinone reductase-activated probes had high specificity and sensitivity for the detection of microtumors and were thus suitable for the evaluation of drug action and efficacy in clinical-related tumor models and preclinical animal studies.

**Fig. 43:**
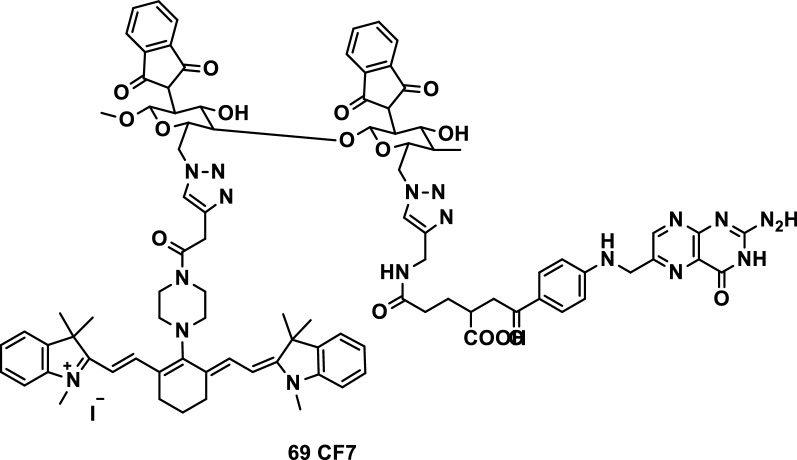
Structure of probe 69

Self-assembled nanoparticles (CF7Ns) of chemically synthesized folate (FA) and heptamethine cyanine-modified chitosan (CF7, Fig. 43, probe 69) were developed for tumor-specific imaging and photodynamic therapy. The spectrum showed that CF7 had a good binding effect between FA and Cy7. The diameter of CF7Ns measured by DLS was approximately 291.6 nm, and the morphology observed by AFM was a filamentous cluster. The targeting effect of CF7Ns on FA receptor-positive HeLa cells was higher than that of non-FA-modified nanoparticles. The cytotoxicity and apoptotic analysis showed that the NIR irradiation of CF7Ns could induce HeLa cell apoptosis and improve the therapeutic effect. The photodynamic mechanism of CF7Ns was confirmed on the basis of the determination of ROS and cytokines related to cell apoptosis. The experiments proved that CF7Ns was a tumor targeting carrier for fluorescence imaging and photodynamic therapy [85].

**Fig. 44:**
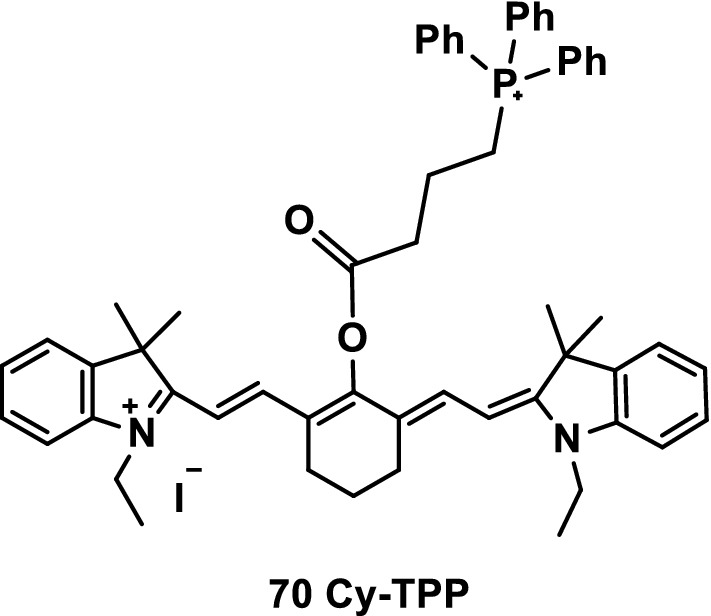
Structure of probe 70

The development of functional drugs with tumor-targeting anticancer activity for tumors detected in imaging determines the future of personalized cancer treatment. However, these functional preparations have not been widely used in clinical practice because of the low efficiency of drug delivery, poor imaging specificity of tumors, development of drug resistance, and safety considerations for potential toxicity. Therefore, the combination of therapeutic drugs and appropriate fluorescent probes is the commonly used strategy for the research and development of these functional drugs. Ning et al. designed and synthesized a mitochondrial-targeted visualization anticancer agent Cy-TPP with absorption and emission curves in the NIR (Fig. 44, probe 70). Subcellular localization and cell viability analysis showed that the probe had mitochondrial-targeted NIR imaging and effective antiproliferation capabilities (IC50 = 3.04 μM). Therefore, Cy-TPP can be used as a new anticancer drug with mitochondrial-targeting ability [86].

**Fig. 45:**
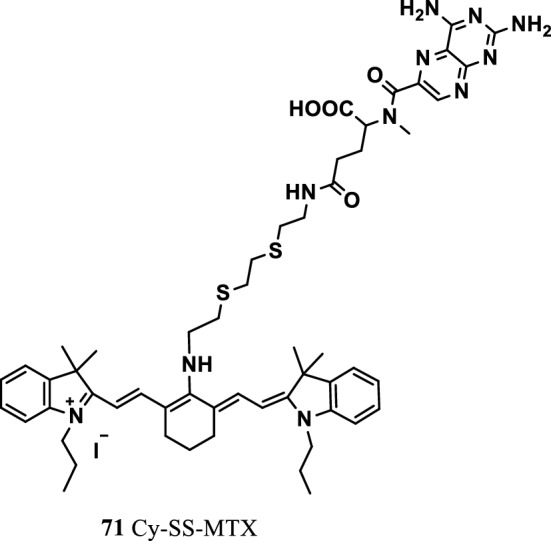
Structure of probe 71

Sanpeng Li’s team synthesized a novel tumor-targeting and GSH-activated conjugate-based theranostic prodrug Cy-SS-methylate (MTX) (Fig. 45, probe 71). The prodrug was constructed by binding disulfide Cy (IR 780) to MTX. The Cy dye as the targeting molecule carried the prodrug into human cancer cells and was activated by high concentrations of GSH in the tumor. The results showed the prodrug’s good capability for targeting tumors. At the same time, the prodrug significantly improved the antitumor ability and immensely reduced the toxicity of free MTX to normal cells. The structure of prodrug Cy exhibited an absorption peak at 654 nm in the UV–vis spectrum. A new UV–vis absorption peak appeared at 802 nm in the cyanide structure of the prodrug when GSH destroyed its disulfide bond. Meanwhile, the fluorescence emission peak of 750 nm (640 nm excitation) changed to 808 nm (745 nm excitation). The PA signals excited at 680 and 808 nm changed. The drug was successfully used in NIR laser excitation and cancer-targeting therapy for fluorescence and PA imaging to track MTX activation in real time. These studies promoted the tumor-targeted heptamethine cyanine dye-based prodrug as a reporting group for targeted therapy and the real-time tracking of drug activation [87].

**Fig. 46:**
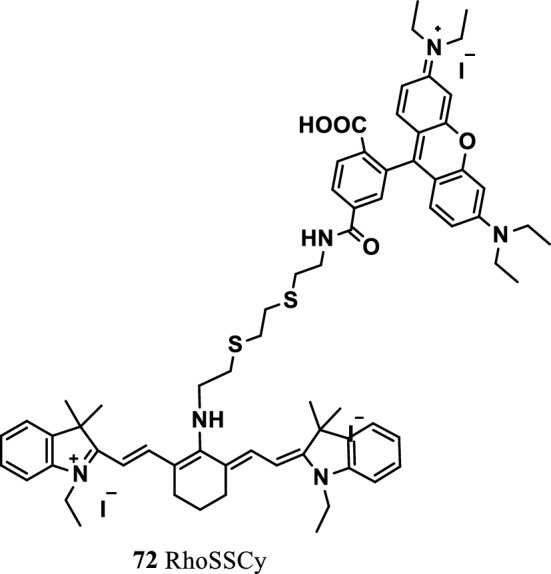
Structure of probe 72

Meng et al. reported a small molecule probe RhoSSCy with sensing, targeting, multimode imaging, and therapeutic functions (Fig. 46, probe 72). The RhoSSCy probe was synthesized from Rho and IR765 (Cy) by combining a disulfide bond and an amino group with adjustable acidity and basicity. The probe quantitatively detected free mercaptan and directly detected the changes of intracellular acidity and alkalinity and mercaptan concentration. At the same time, RhoSSCy molecules easily entered and accumulated in the tumor cells, indicating its NIRF/PA dual-mode imaging and the anticancer effect of high-efficiency photodynamic therapy in vivo and in vitro. Overcoming the shortcomings of NIRF dyes combined with exotic thermal analysis technology, this well-defined small-molecule probe laser was deemed suitable for multimode imaging and could offer important application value for targeted molecular diagnosis and imaging treatment of cancer [88].

**Fig. 47:**
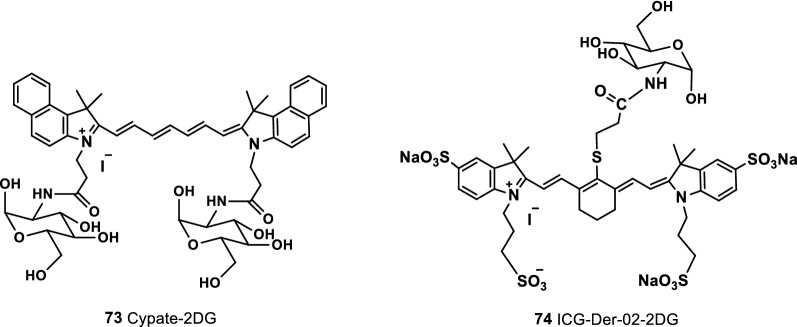
Structures of probes 73 and 74

Luo et al. [89] investigated various response mechanisms of tumor cells and observed that that differences in the energy metabolism of tumor cells could be used as therapeutic targets for hyperglycolysis. Guo et al. [90] used tumor cells to consume more glucose in the body than normal cells to synthesize two fluorescent probes Cypate-2DG and ICG-Der-02-2DG (Fig. 47, probes 73 and 74). The tumor targets of the two NIR fluorescence probes were compared through NIR fluorescence imaging, and the results showed that ICG-Der-02-2DG was more soluble in water than Cypate-2DG and was easily removed by the kidneys. In cancer cells, a correlation was found between the targeting ability of the probe and the level of glucose transporter protein expression. Cypate-2DG and ICG-Der-02-2DG were found to have a tumor-targeting ability, but Cypate-2DG showed a stronger tumor-targeting ability and was proportional to the glucose transporter. The two probes can be widely used in the optical imaging of tumors and glucose-related diseases.

**Fig. 48:**
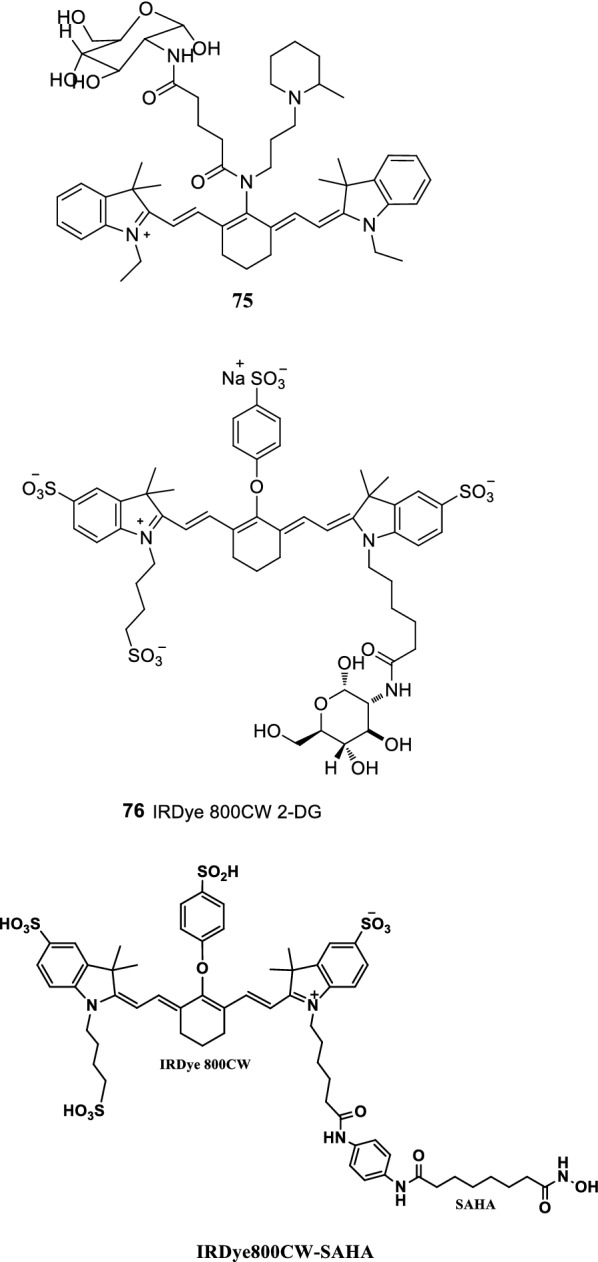
Structures of probes 75, 76 and IRDye800CW-SAHA

Vendrell et al. reported an NIR fluorescence glucose derivative (probe 75) (Fig. 48). The absorption rate of the probe in cancer cells was significantly higher than that in normal cells. At the same time, the probe had a stronger labeling ability for cancer cells than the NIRF dye IRDye-800CW2-DG (Fig. 48, probe 76) [91].

Histone deacetylases (HDACs) were found to be highly expressed in tumor samples from patients with Hepatocellular carcinoma (HCC), and suberoylanilide hydroxamic acid (SAHA) was clinically demonstrated to be a potent tumor suppressor for HCC. Accordingly, in this study, researchers synthesized HDAC-targeted NIR fluorescence probes for fluorescent imaging of HCC by using SAHA, which had a high affinity to HDAC, as a targeting group. In vitro experiments, SAHA was labelled with fluorescein isothiocyanate (FITC) to test its targeting, and the results showed that FITC-SAHA was specifically absorbed by HCC Bel-7402 cells. In vivo experiments, near infrared fluorescence dye IRDye800CW-SAHA on subcutaneous and in situ HCC tumor model in mice showed a strong targeted fluorescence imaging. IRDye800CW-SAHA could successfully guide surgical resection of hepatocellular carcinoma in situ and non-toxic to healthy tissue and cells. The results showed that the IRDye800CW-SAHA was a fluorescent probe for liver cancer diagnosis and surgery navigation. [92].

**Fig. 49:**
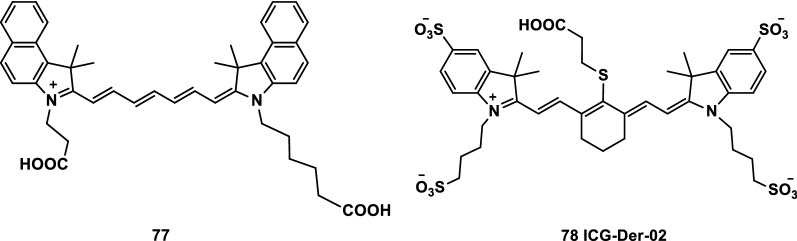
Structures of probes 77 and 78

Cholesterol is the main component of the cell membrane, and cancer cells require more cholesterol to proliferate than normal cells. Thus, cancer patients frequently exhibit symptoms such as low cholesterol. Low-density lipoprotein (LDL), which mainly stores cholesterol in human plasma, enters the cells mostly through receptor mediation. Therefore, the relationship of LDL receptors and tumors has been widely investigated in cancer cell research [93]. Deng et al. [94] used NIR fluorescence probes 77 and ICG-Der-02 (Fig. 49, probes 77 and 78) as structural cores and designed and synthesized the NIR organic fluorescent probes FA-PEG-ICG-Der-01 and LDL-ICG-Der-02. The fluorescence signal intensity and light stability of the synthesized FA-PEG-ICG-Der-01 and LDL-ICG-Der-02 probes were higher than those of the corresponding dye monomers. The results of in vivo targeting imaging in mice showed that the two probes could identify the biological activity of folic acid and LDL. The two probes accurately targeted relevant tumor tissues, with clear fluorescence imaging and metabolization. The probes can be used for the early diagnosis and localization of tumors.

**Fig. 50:**
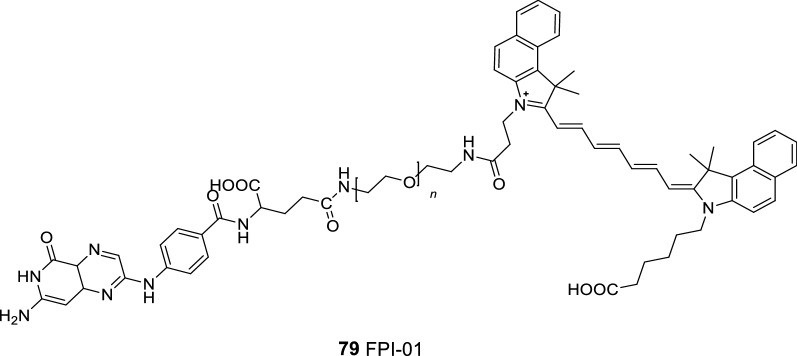
Structure of probe 79

The FA receptor (FR) is an important means for cells to acquire FA. The expression level of FR in many malignant tumors is higher than that in normal cells. Therefore, FA (the corresponding ligand of folic acid receptor) has become an important target in the treatment of malignant tumors [95]. Liu et al. [96] synthesized a fluorescent probe FPI-01 (probe 79) by linking FA with benzoindole heptamethyocyanine dye 77 (Fig. 49) and PEG (Fig. 50). The results showed that the probe has high affinity to the FA receptor without obvious cytotoxicity and metabolic excretion in vitro. The probe has great potential in the diagnosis of FA receptor-positive tumors.

**Fig. 51:**
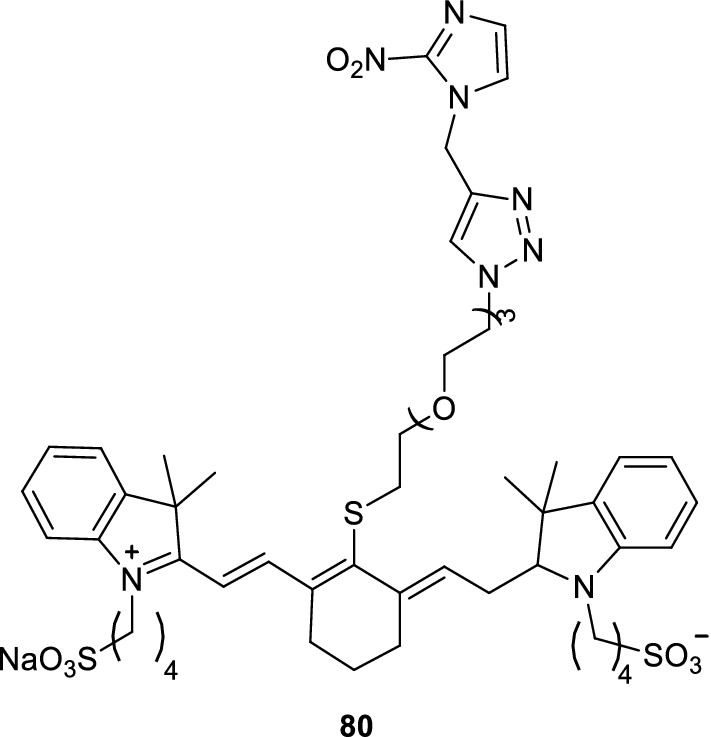
Structure of probe 80

Anaerobic characteristics are a prominent feature of malignant tumors. Tumor cells at different anaerobic levels have different effects on the response of tumor cells to normal chemotherapy, radiotherapy, and other nonsurgical treatments. Thus, a noninvasive imaging technology utilizing the anaerobic characteristics of malignant tumors should be developed. Youssif et al. [97] designed and synthesized a corporate probe (probe 80) for the fluorescence imaging of malignant tumors (Fig. 51). In the experiment, the fluorescence signal intensity of hypoxic cells was significantly higher than that of normal cells with the introduction of probe 80 to pancreatic cancer cells. The results showed that the fluorescence intensity of the probe in cancer cells was closely related to the anaerobic activity of cancer cells. After 6 h of injection, the fluorescence signal of the probe in the tumor significantly decreased and gradually disappeared, indicating that some structural changes and instability occurred in the anaerobic tumor cells. Thus, the probe was wrapped with nanoparticles to improve its instability to anaerobic tumors. This process prolonged the interaction of drugs and blood and significantly reduced the nonspecific accumulation of drugs in the biological system. Thus, the probe plays a role in the detection of long-term treatment.

**Fig. 52:**
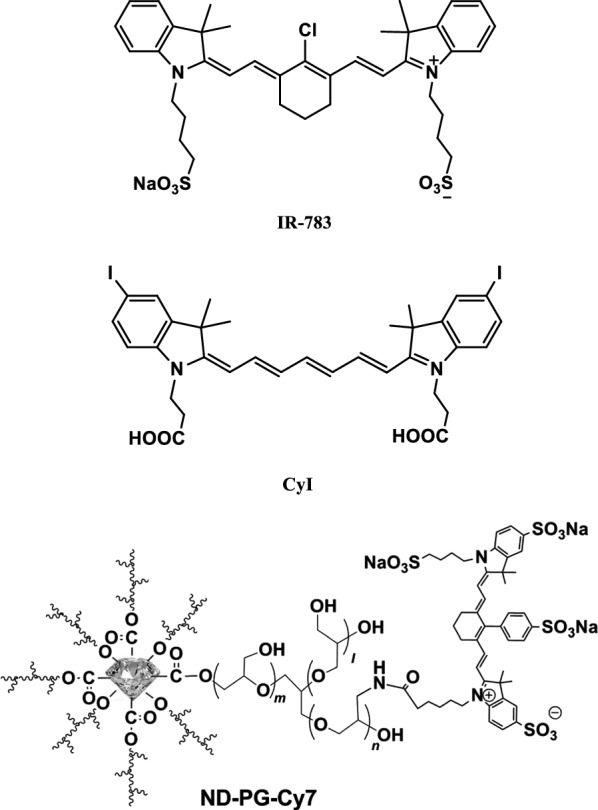
Structures of probes IR-783, CyI, and ND-PG-Cy7

Duan et al. developed a probe IR-783 (Fig. 52) as a rapid imaging agent to detect human cervical cancer. They investigated the uptake, accumulation, and subcellular localization of IR-783 dyes in cervical cancer cells. Mouse whole-body imaging was conducted to detect the specific staining absorption and retention of human cervical cancer xenografts and freshly collected clinical cervical cancer specimens. Frozen tissue sections were used to determine the accumulation of dyes at the tissue and cell levels. Circulating tumor cells containing cervical cancer cells in peripheral blood were detected. The results showed that IR-783 can be specifically uptaken by cultured cervical cancer cells, human cervical cancer xenotransplantation, human cervical cancer cells, and human cervical cancer tissues but not by normal cell tissues. This study demonstrated the ability of IR-783 to detect cervical cancer cells in clinical specimens and circulating blood. The probe can be used in clinical practice as a modal agent for deep tissue imaging of cervical cancer. [98].

Cao et al. [99] reported a novel NIR probe CyI using heavy atom iodine and ICG derivative Cy7 (Fig. 52). The probe had the ability to increase the production and heat production of ROS in vivo, and its fluorescence properties were stable, which could be used for non-invasive fluorescence imaging in vivo. In vitro and in vivo experiments showed that CyI could rapidly and simultaneously produce enhanced ROS and heat under NIR irradiation, inducing apoptosis of HepG2 tumor cells. The results indicated that the probe was an ideal deep tumor therapy agent for the rapid cooperative photodynamic therapy (PDT)/photothermal therapy (PTT) treatment of deep tumor tissue.

Nanoparticles have been reported to achieve preferential accumulation in tumors by combined effects of active and passive targeting. Herein, the researchers synthesized a polyglycerol-functionalized nanodiamonds (ND-PG), which was then combined with a cyanine dye (Cy7) to form ND-PG-Cy7. It was found that ND-PG-Cy7 could accumulate in the tumor first, and the fluorescence images in vivo and in vitro showed clear fluorescence. One possible reason was that ND-PG-Cy7 had a longer in vivo blood circulation time (half-life: 58 h determined by the pharmacokinetic analysis) [100].

### Application of NIR Fluorescence probes to other fields

Indocyanine dyes can be used to detect nucleic acids, proteins, and antibodies. The convenient labeling of proteins is important to observe their functions under physiological conditions. In tissues, the value of heptadecylamine cyanide dyes (Cy-7) is obvious because they are absorbed in the NIR with large light penetration.

**Fig. 53:**
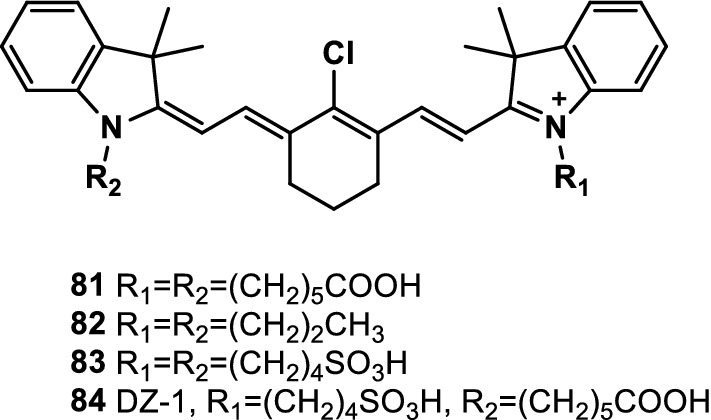
Structures of probes 81–84

Lin et al. [101] observed that Cy-7 dyes contain meso-Cl and can covalently bind to free Cys residues under physiological conditions (water environment, near-neutral pH, and 37 °C). The results showed that the meso-Cl of the dye was replaced by free thiols in the protein. Moreover, the nucleophilic side chains in amino acids, such as Tyr, Lys, and Ser, did not react. This condition showed the feasibility of using NIR fluorescence probes for the convenient and selective labeling of proteins. The probe MHI-148 (Fig. 53, Probe 81) was used to label free Cys residues, such as vimentin and serum albumin [102]. Other Cy-7 dyes containing meso-Cl (Fig. 53, probes 82, 83, and 84) were used to label vimentin. This process could be applied to the combination of NIR dyes containing meso-Cl with antibodies, monomers, and nanoparticles to form selective reagents for in vivo optical imaging. This method avoided dye modification to include the functions of maleimide or succinimide compared with traditional conjugation techniques [103, 104].

Coline Canovas’ team proved that probe IR-783 (Fig. 53, probe 83) containing a chloro-cyclohexyl moiety within its polymethine chain can react selectively at neutral pH, with Cys residues in proteins providing stable, site-specifically labeled conjugates that are emitted in the NIR spectral window. An example of this reaction was the labeling of polypeptides and two protein models, namely, albumin and Fab’ antibody fragments. As shown in the mouse model, the obtained fluorescent protein was stable and suitable for NIR fluorescence imaging in vivo. This simple step did not require the prior derivatization of the fluorophore with a biological conjugated handle and might promote the production and use of near-infrared-labeled proteins in life sciences [105].

**Fig. 54:**
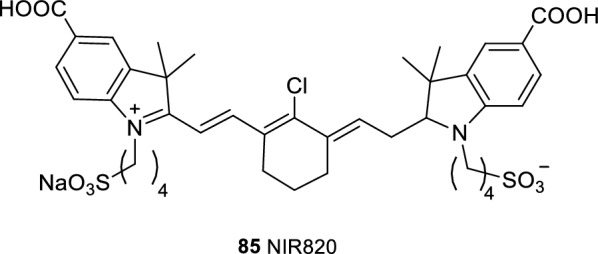
Structure of probe 85

Hong et al. [106] reported the determination of nucleic acid using a new indoxymethachene NIR fluorescence cyanine dye at the range of 0.08–1.2 g/mL. James et al. [107] synthesized an NIRF cyanine dye using succinimide ester and isocyanate as the recognition group and cyanine dye as the fluorophore. The experimental results showed that the dye has good physiological activity. Pham et al. [108] synthesized probe NIR820 (Fig. 54, probe 85) with good stability and fluorescence, and involved a simple operation, entailed low costs, and enabled the easy purification.

**Fig. 55:**
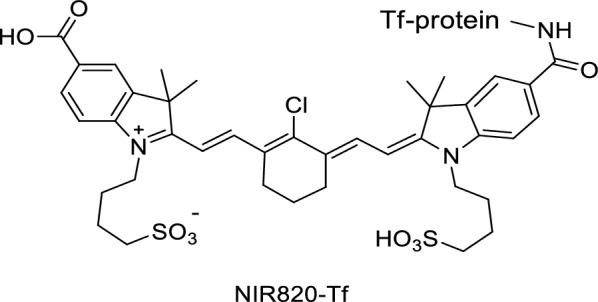
Structure of probe NIR820-Tf

A novel NIR fluorescence probe NIR820-Tf was generated by combining TfR with NIR820 (Fig. 55). Fluorescence signals were observed in the tumor cells with the introduction of the probe in the gliosarcoma cell culture for 1 h. This result indicated that the NIR fluorescence-labeled Tf was absorbed by the cells and that NIR820-Tf did not hinder the absorption of Tf by gliosarcoma cells. Experiments showed that the dye NIR820, which was not bound to Tf, did not penetrate the cell membrane of gliosarcoma and must be metabolized and excreted from the body. The integrin ανβ_3_ receptor is not expressed in normal tissues but is overexpressed in many tumor cells. Thus, the integrin ανβ_3_ receptor can be used as a target for the diagnosis of tumor cells. Houston et al. [109] synthesized a new NIR fluorescence probe NIR820-Tf and labeled it with the radioactive isotope ^III^In and IRDye800 integrin ανβ_3_ for the detection of melanoma.

MMPs are a group of proteases that degrade the extracellular matrix and other extracellular proteins. Inactive MMPs are metabolized to the cell membrane or extracellular proteins. The active sites can be observed in the catalytic region through the hydrolysis and removal of the prepeptide region, thus activating the MMPs. Studies have shown that MMPs, including MMP-2 and MMP-9, are highly expressed in various tumor cells. Akers et al. [110] synthesized an NIR fluorescence probe LS276-THP based on trihelicopeptide to detect cancer-related MMPs in vivo. The fluorescence signal of the probe was enhanced, and the fluorescence quantum yield of the probe increased by approximately four times when trihelicopeptide was hydrolyzed, catalyzed, and activated and then released six labeled peptide chains by MMPs. After the self-assembly of the three-helix structure, the fluorophores were relatively close to one another because of the intertwined peptide chains, resulting in fluorescence quenching and reduction of the corresponding fluorescence intensity. LS276-THP was injected into tumor-inoculated mice, and the detection showed that the fluorescence signal in the tumor tissue was stronger than that in normal muscle tissue. Subsequently, Ilomastat, an MMP inhibitor, was injected into the above experimental mice, and the fluorescence signal intensity was found to be weakened. The results showed that LS276-THP was suitable for the detection of tumor-related MMP-2 and MMP-9 in vivo.

**Fig. 56:**
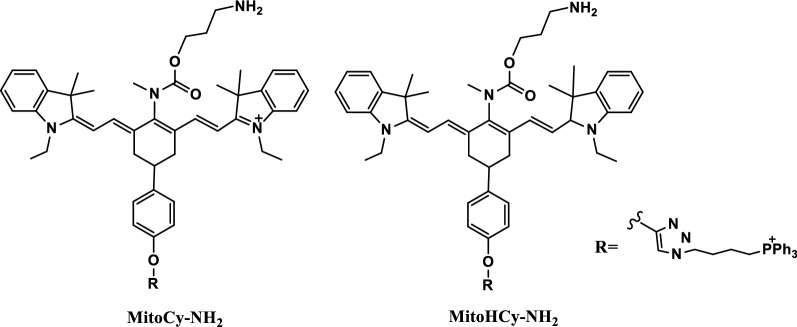
Structures of probes MitoCy-NH_2_ and MitoHCy-NH_2_

Monoamine oxidase (MAOs) is a novel biomarkers, including MAO-A and MAO-B isoforms. It plays an important role in maintaining the homeostasis of biogenic amines via catalyzing the oxidation of biogenic amines to generate the corresponding aldehydes and ROS. MAO-A is thought to be associated with neuropsychiatric and depressive disorders, while MAO-B is thought to be associated with several neurodegenerative. Therefore, in order to investigate their different roles in different diseases, it is essential to selectively detect changes in MAOs. Two novel NIR fluorescence probes, MitoCy-NH_2_ and MitoHCy-NH_2_ (Fig. 56), were synthesized to provide synergistic imaging of MAO-B and its contribution to oxidative stress in cells and in mice aging models. These probes were composed of heptamethine cyanine, propanamide and triphenylphosphonium cation. In the replicative senescence model, MitoHCy-NH_2_ had the ability to synergistically detect MAO-B and its response to oxidative stress. And the probe MitoCy-NH_2_ could offer ratiometric NIR fluorescence for the selective detection of MAO-B in the H_2_O_2_-induced cell aging model and in mice aging models. The excellent detection performance of these probes maked them useful chemical tools for selective analysis of MAO-B and its contribution to oxidative stress in biological systems [111].

## Conclusions

With the rapid development of optical detection and imaging technology, researchers have widely investigated the use of NIRF dyes as fluorescent probes and indicators. In this study, the research progress of indole heptamethylene cyanine dyes was reviewed in terms of the structure, properties, and biological applications of NIR fluorescence probes to provide reference for the future development and research of novel indole heptamethylene cyanine dyes.

Two important advantages of indole heptamethylene cyanine dyes are their small self-fluorescence and the background absorption of the tissue itself. However, the application of indole heptamethylene cyanine dyes is hindered because of the lack of fluorophores with high light stability and high fluorescence efficiency. Few indole heptamethylene cyanine dyes that are applicable to biology have been widely used in commercial applications. Many new indole heptamethylene cyanine dyes with good fluorescence performance have been developed to meet the biological detection requirements of fluorescent probes. Different groups should be introduced in the dye structure, and the stability and fluorescence efficiency of dyes can be improved by changing their molecular structure with different biological detection functions. Indole heptamethylene cyanine dyes with low background, strong light stability, high fluorescence quantum yield, and easy purification should be developed.

## Data Availability

Data supporting our findings is contained within the manuscript; any additional data will be shared upon request to the corresponding author.

## Abbreviations

NIR: Near-infrared
NIRF: Near-infrared fluorescent
PTT: Photothermal therapy
PY: N1-(pyridine-4-methyl) ethane-1,2-diamine
PA: Photoacoustic
PEG: Polyethylene glycol
ICG: Indocyanine green
SCy-OHs: Tautomorphic isomer
DIPCY: Dipicolylcyanine
DPEN: Dipicolylethylenediamine
H_2_S_n_: Hydrogen polysulfide
ROS: Reactive oxygen species
H_2_O_2_: Hydrogen peroxide
O_2_^·−^: Superoxide anion radical
HO·: Hydroxyl radical
ONOO^−^: Peroxynitrite
NO: Nitric oxide
GSH: Glutathione
AAs: Amino acids
Sec: Selenocysteine
UV-vis: Ultraviolet-visible
hNQO1: Quinone oxidoreductase isozyme I
FA: Folate
MTX: Cy-SS-methylate
HDACs: Histone deacetylases
HCC: Hepatocellular carcinoma
SAHA: Suberoylanilide hydroxamic acid
FITC: Fluorescein isothiocyanate
LDL: Low-density lipoprotein
FR: Folate receptor
PDT: Photodynamic therapy
PTT: Photothermal therapy
ND-PG: Polyglycerol-functionalized nanodiamonds
MAOs: Monoamine oxidase
